# An autophagy enhancer ameliorates diabetes of human *IAPP*-transgenic mice through clearance of amyloidogenic oligomer

**DOI:** 10.1038/s41467-020-20454-z

**Published:** 2021-01-08

**Authors:** Jinyoung Kim, Kihyoun Park, Min Jung Kim, Hyejin Lim, Kook Hwan Kim, Sun-Woo Kim, Eun-Seo Lee, Hyongbum (Henry) Kim, Sung Joo Kim, Kyu Yeon Hur, Jae Hyeon Kim, Jin Hee Ahn, Kun-Ho Yoon, Ji-Won Kim, Myung-Shik Lee

**Affiliations:** 1 Severance Biomedical Science Institute, Yonsei University College of Medicine, Seoul, Korea; 2 Department of Internal Medicine, Yonsei University College of Medicine, Seoul, Korea; 3 Department of Health Sciences and Technology, Samsung Advanced Institute for Health Science and Technology, Sungkyunkwan University School of Medicine, Seoul, Korea; 4 Department of Endocrinology and Metabolism, College of Medicine, The Catholic University of Korea, Seoul, Korea; 5 Department of Pharmacology, Yonsei University College of Medicine, Seoul, Korea; 6 Transplantation Center, Sungkyunkwan University School of Medicine, Seoul, Korea; 7 Department of Surgery, Sungkyunkwan University School of Medicine, Seoul, Korea; 8 Department of Medicine, Samsung Medical Center, Sungkyunkwan University School of Medicine, Seoul, Korea; 9 Department of Chemistry, Gwangju Institute of Science and Technology, Gwangju, Korea

**Keywords:** Macroautophagy, Type 2 diabetes

## Abstract

We have reported that autophagy is crucial for clearance of amyloidogenic human IAPP (hIAPP) oligomer, suggesting that an autophagy enhancer could be a therapeutic modality against human diabetes with amyloid accumulation. Here, we show that a recently identified autophagy enhancer (MSL-7) reduces hIAPP oligomer accumulation in human induced pluripotent stem cell-derived β-cells (hiPSC-β-cells) and diminishes oligomer-mediated apoptosis of β-cells. Protective effects of MSL-7 against hIAPP oligomer accumulation and hIAPP oligomer-mediated β-cell death are significantly reduced in cells with knockout of MiTF/TFE family members such as *Tfeb* or *Tfe3*. MSL-7 improves glucose tolerance and β-cell function of *hIAPP*^+^ mice on high-fat diet, accompanied by reduced hIAPP oligomer/amyloid accumulation and β-cell apoptosis. Protective effects of MSL-7 against hIAPP oligomer-mediated β-cell death and the development of diabetes are also significantly reduced by β-cell-specific knockout of *Tfeb*. These results suggest that an autophagy enhancer could have therapeutic potential against human diabetes characterized by islet amyloid accumulation.

## Abstract (web summary)

Islet amyloid polypeptide (IAPP) deposition is associated with islet cell loss in diabetes. Here the authors show that a small molecule autophagy enhancer reduces IAPP accumulation in vitro, and also improves glucose tolerance in *hIAPP*^+^ mice fed high-fat diet, accompanied by reduced hIAPP accumulation, in vivo.

## Introduction

While most studies on the basic pathogenic mechanism of diabetes employ mouse models in which genetic modulation is possible, human diabetes and mouse diabetes are different in several key features. For instance, the structure and function of pancreatic islets are different between mouse models and humans^1^. Such differences may be responsible for the failure of antidiabetic drugs developed using mouse models in human clinical trials. One of the most intriguing differences between mouse models of diabetes and human diabetes is the deposition of amyloid in islets of human patients with diabetes but not in those of mouse models. Islet amyloid deposition is observed in over 90% of human diabetes patients but not in murine diabetes, which is due to the differences in the amino acid sequences of the islet amyloid polypeptide (IAPP)^2^. Others and we previously reported that human-type IAPP (hIAPP) oligomer or amyloid accumulation in transgenic mice expressing hIAPP (*hIAPP*^+^ mice) or *hIAPP* knock-in mice in which endogenous murine *IAPP* gene was replaced by *hIAPP* markedly increases when β-cell autophagy is deficient, suggesting that autophagy is critical in the clearance of hIAPP oligomer or amyloid^3–5^. These data are consistent with a proposition that aggregate or amyloid-prone proteins are preferentially cleared by autophagy rather than proteasomal degradation^6^. These results also suggest the possibility that autophagy enhancers may be employed as a therapeutic modality against human diabetes characterized by islet amyloid accumulation. Indeed, we have observed that trehalose that has been reported to have autophagy-enhancing activity^7^ could ameliorate metabolic profile of *hIAPP*^+^ mice fed high-fat diet (HFD). However, trehalose has been developed as a chaperoning agent or hydrating agent, and its autophagy enhancing activity has been questioned in a recent paper^8^.

We recently identified and developed bona fide autophagy enhancer small molecules in a high-throughput screening of a chemical library that can have beneficial metabolic effects in obese mouse models by enhancing clearance of lipid and ameliorating metabolic inflammation. These effects were attributed to calcineurin-dependent dephosphorylation and activation of TFEB that belongs to the MiTF/TFE family and is a master regulator of lysosome biogenesis and autophagy gene expression^9^. Here, we studied whether our autophagy enhancer small molecule, MSL-7, could expedite clearance of hIAPP oligomer in pancreatic islets and protect β-cells in transgenic mice showing hIAPP accumulation. We found that MSL-7 improved the glucose tolerance and β-cell function of *hIAPP*^+^ mice rendered diabetic by HFD feeding, which was accompanied by reduced accumulation of hIAPP oligomer and islet amyloid. These results suggest that an autophagy enhancer acting in a TFEB-dependent manner could have a therapeutic potential against human diabetes characterized by islet amyloid accumulation.

## Results

### Dephosphorylation and nuclear translocation of TFEB and TFE3 by autophagy enhancer, MSL-7

**Fig. 1:**
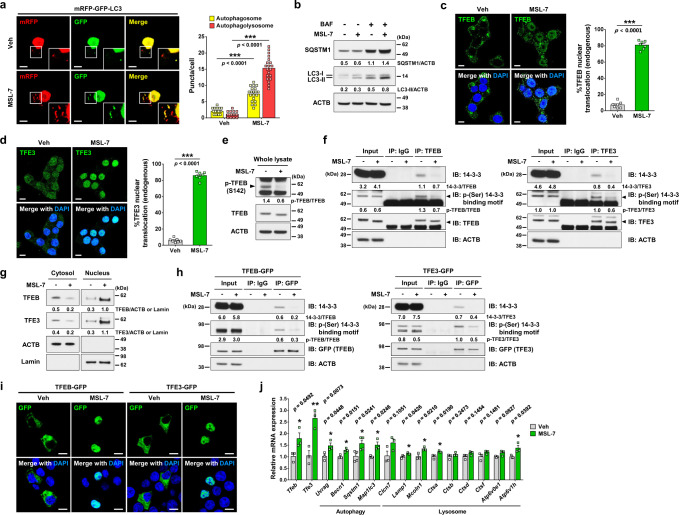
Activation of TFEB/TFE3 by MSL-7. **a** INS-1 cells were transfected with *mRFP-GFP-LC3*. The numbers of yellow (autophagosomes) and red puncta (autophagolysosomes) were counted (*t* = 9.8, df = 38 for autophagosome; *t* = 17.2, df = 38 for autophagolysosome) (right). Representative pictures are presented (left). Inset images were magnified. **b** INS-1 cells were treated with MSL-7 in the presence or absence of bafilomycin A1 (BAF). Immunoblotting using the indicated antibodies (ACTB, β-actin) were conducted. Numbers indicate fold changes normalized to ACTB bands. **c**–**d** Nuclear translocation of TFEB (**c**) and TFE3 (**d**) by MSL-7 was counted (*t* = 29.9, df = 12 in **c**; *t* = 38.7, df = 12 in **d**) (right of **c**, **d**). Representative pictures are shown (left of **c**, **d**). **e** INS-1 cells were treated with MSL-7, and immunoblotting using the indicated antibodies were conducted. Numbers indicate fold changes normalized to total TFEB bands. **f** After lysis of INS-1 cells treated with MSL-7 for 4 h, immunoprecipitation (IP) was conducted using anti-TFEB (left) or anti-TFE3 antibody (right). Supernatant was subjected to immunoblot analysis (IB) using the indicated antibodies. Numbers indicate fold changes normalized to total TFEB or TFE3 bands. **g** Nuclear and cytosolic fractions of lysates from INS-1 cells treated with MSL-7 were subjected to immunoblot analysis using the indicated antibodies. Numbers indicate fold changes normalized to ACTB bands (cytosol) or Lamin bands (nuclear). **h** After lysis of *Tfeb-GFP-*transfected (left) or *Tfe3-GFP-*transfected cells (right) treated with MSL-7, IP using anti-GFP antibody was conducted. **i** After treatment of *Tfeb-GFP-*transfected or *Tfe3-GFP*-transfected cells with MSL-7, confocal microscopy was conducted. Numbers indicate fold changes normalized to GFP bands. **j** Real-time RT-PCR was performed using mRNA from INS-1 cells treated with MSL-7 for 6 h and specific primers. All data in this figure are the means ± SEM from more than 3 independent experiments performed in triplicate. (scale bar, 5 μm) *P < 0.05; **P < 0.01; ***P < 0.001 by two-tailed Student’s *t*-test (**a**, **c**, **d**, **j**). Source data are provided as a Source Data file.

We first studied whether MSL-7 can induce autophagy in INS-1 insulinoma cells. Confocal microscopy after transfection of INS-1 cells with *mRFP-GFP-LC3* tandem construct showed that the numbers of both autophagosomes (yellow puncta) and autophagolysosomes (red puncta) were significantly increased after treatment with MSL-7 (Fig. 1a), indicating that MSL-7 induces autophagic activity in INS-1 cells. Conversion of LC3-I to LC3-II in the presence of bafilomycin A1was also increased by MSL-7 (Fig. 1b and Supplementary Fig. 1a), supporting increased autophagic flux by MSL-7. Since TFEB, a member of MiTF/TFE family, is a master regulator of autophagy gene expression and lysosomal biogenesis^10^ and TFEB is expressed in primary murine islet cells (Supplementary Fig. 2a, b), we studied TFEB activation in INS-1 cells. As expected, a significant increase of the number of INS-1 cells with nuclear TFEB was observed after treatment with MSL-7 (Fig. 1c), indicating that MSL-7 activates autophagy of INS-1 cells through TFEB nuclear translocation. Probably because of TFEB activation, expression of SQSTM1 (also known as p62), a target of TFEB^11^, was not reduced but increased by MSL-7 despite activation of autophagy (Fig. 1b and Supplementary Fig. 1a). We next studied TFE3, another member of the MiTF/TFE family regulating autophagy gene expression and lysosomal biogenesis^12^, which is also expressed in primary murine islets (Supplementary Fig. 2a, b). Translocation of TFE3 was also well observed after MSL-7 treatment of INS-1 cells (Fig. 1d). Since the phosphorylation status of MiTF/TFE family members is critical in nuclear translocation and induction of their target genes, we studied phosphorylation of MiTF/TFE family members. When we studied phosphorylation of S142 of TFEB, one of the most important phosphorylation sites of TFEB^13^, S142 phosphorylation was markedly reduced by MSL-7 (Fig. 1e and Supplementary Fig. 1b), which was consistent with previous data using other types of cells^9^. We also studied phosphorylation of S211 of TFEB, another important phosphorylation site of TFEB, using immunoprecipitation assay based on the binding of the phospho-S211 motif of TFEB to 14-3-3 protein^14^. Band intensity of 14-3-3 protein identified by immunoblotting with anti-14-3-3 antibody in TFEB immunoprecipitate, thus TFEB-bound 14-3-3 protein, was markedly reduced by MSL-7 (Fig. 1f and Supplementary Fig. 1c), indicating decreased phosphorylation of S211 of TFEB by MSL-7. Band intensity of TFEB with 14-3-3 binding motif identified by immunoblotting with anti-phospho-(Ser) 14-3-3 binding motif antibody in TFEB immunoprecipitate, thus phospho-S211-TFEB^14^ was also markedly reduced by MSL-7 (Fig. 1f and Supplementary Fig. 1c), again supporting decreased phosphorylation of S211 of TFEB by MSL-7. We next studied the phosphorylation of TFE3 using a similar immunoprecipitation assay based on the binding of phospho-S321 motif of TFE3 to 14-3-3 protein^12^. Band intensity of 14-3-3 protein identified by immunoblotting with anti-14-3-3 antibody in TFE3 immunoprecipitate, thus TFE3-bound 14-3-3 protein, and that of TFE3 with 14-3-3 binding motif identified by immunoblotting with anti-phospho-(Ser) 14-3-3 binding motif antibody in TFE3 immunoprecipitate, thus phospho-S321-TFE3^12^, were notably reduced by MSL-7 (Fig. 1f and Supplementary Fig. 1c). Markedly increased nuclear translocation of TFEB and TFE3 likely due to reduced phosphorylation upon treatment of INS-1 cells with MSL-7 was confirmed by immunoblot analysis after nuclear fractionation (Fig. 1g and Supplementary Fig. 1d). Reduced phosphorylation of S211-TFEB or S321-TFE3 by MSL-7 was also observed when *Tfeb-GFP* or *Tfe3-GFP* was overexpressed in INS-1 cells using the same immunoprecipitation assay employing anti-GFP antibody (Fig. 1h and Supplementary Fig. 1e). Consequently, nuclear translocation of TFEB-GFP or TFE3-GFP was markedly increased after MSL-7 treatment (Fig. 1i), similar to the nuclear translocation of endogenous TFEB or TFE3. Treatment with MSL-7 inducing nuclear translocation of TFEB and TFE3 upregulated expression of several autophagy genes and lysosomal genes downstream of MiTF/TFE family members such as *Uvrag*, *Becn 1*, *Sqstm1*, *Map1lc3*, *Lamp1*, *Mcoln1*, *Ctsa,* or *Atp6v1h* (Fig. 1j and Supplementary Table 1). Expression of *Tfeb* or *Tfe3* themselves was also induced by MSL-7 (Fig. 1j).

### Expedited clearance of hIAPP oligomer by MSL-7

**Fig. 2:**
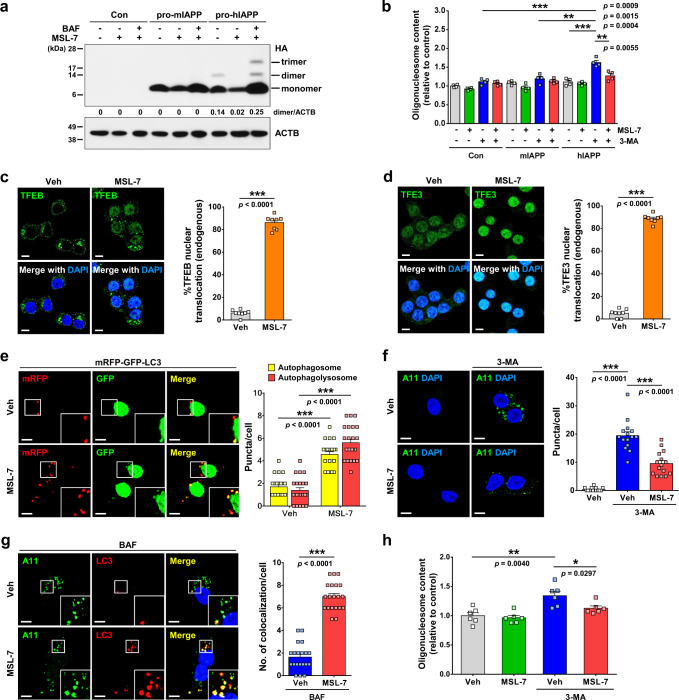
hIAPP/sIAPP oligomer clearance by MSL-7. **a** *Prepro-mIAPP-HA-*transfected or *Prepro-hIAPP-HA*-transfected INS-1 cells were treated with MSL-7 in the presence or absence of bafilomycin A1 (BAF). Lysate was subjected to immunoblotting using anti-HA or anti-β-actin antibody (ACTB). Numbers indicate fold changes of hIAPP dimer normalized to ACTB bands. (Con, control-transfected). **b** *Prepro-mIAPP-HA-*transfected or *Prepro-hIAPP-HA*-transfected cells were treated with MSL-7 in the presence or absence of 3-MA. Oligonucleosome content was determined (*F* = 19.5, df treatment = 11, df residual = 36). **c–d** MSL-7-induced nuclear translocation of TFEB (**c**) and TFE3 (**d**) in monkey islet cells was counted (*t* = 33.2, df = 14 in **c**; *t* = 45.7, df = 14 in **d**) (right of **c**, **d**). Representative pictures are presented (left of **c**, **d**). **e** *mRFP-GFP-LC3*-transfected monkey islet cells were treated with MSL-7. The numbers of autophagosome (*t* = 9.5, df = 44) and autophagolysosome (*t* = 10.9, df = 44) were counted (right). Representative pictures are presented (left). Inset images were magnified to show red (autophagolysosomes) and yellow (autophagosomes) puncta. **f** Monkey islet cells treated with MSL-7 in the presence or absence of 3-MA were subjected to immunostaining using A11 antibody. The number of A11^+^ puncta was counted (*F* = 86.3, df treatment = 2, df residual = 42) (right). Representative pictures are presented (left). **g** Monkey islet cells treated with MSL-7 in the presence of BAF, cells were immunostained using A11 and anti-LC3 antibodies. The number of LC3 puncta colocalized with A11^+^ oligomer was counted (*t* = 13.9, df = 38) (right). Representative pictures are shown (left). Inset images were magnified to shows LC3 puncta colocalized with A11^+^ oligomer. **h** Apoptosis was determined in MSL-7-treated monkey islet cells in the presence or absence of 3-MA (*F* = 10.1, df treatment = 3, df residual = 20). All data in this figure are the means ± SEM from more than 3 independent experiments performed in triplicate. (scale bar, 5 μm) *P < 0.05; **P < 0.01; ***P < 0.001 by one-way ANOVA with Tukey’s test (**b**, **f**, **h**) and two-tailed Student’s *t*-test (**c**, **d**, **e**, **g**). Source data are provided as a Source Data file.

We previously reported that pro-human IAPP (hIAPP) dimer or trimer accumulates when *prepro-hIAPP* encoding amyloidogenic hIAPP is expressed in INS-1 insulinoma cells^3^ which could be the initial seed for hIAPP oligomer, fibril or amyloid^15,16^. Thus, we studied whether MSL-7 could reduce the pro-hIAPP dimer or trimer accumulation. When INS-1 cells were transfected with *prepro-hIAPP-HA*, pro-hIAPP dimer was observed in addition to hIAPP monomer (Fig. 2a and Supplementary Fig. 1f), consistent with a previous report^3^. When INS-1 cells were transfected with *prepro-murine IAPP* (*mIAPP*)-*HA*, only mIAPP monomer was observed, as expected. When INS-1 cells were incubated with MSL-7, accumulation of the pro-hIAPP dimer following transfection with *prepro-hIAPP-HA* was notably decreased compared to control cells (Fig. 2a and Supplementary Fig. 1f). Decreased accumulation of pro-hIAPP dimer by MSL-7 was reversed by bafilomycin A1 inhibiting lysosomal V-ATPase (Fig. 2a and Supplementary Fig. 1f), suggesting that enhanced autophagic activity or lysosomal proteolysis is responsible for reduced pro-hIAPP dimer accumulation. In addition to pro-hIAPP dimer, pro-hIAPP trimer was also observed in the presence of bafilomycin A1(Fig. 2a and Supplementary Fig. 1f), consistent with an important role of constitutive autophagy in the clearance of pro-hIAPP dimer and trimer^3^. We also studied whether MSL-7 can ameliorate cell death by hIAPP oligomer accumulation which can be augmented by 3-MA increasing hIAPP oligomer accumulation^3^. When INS-1 cells were incubated with MSL-7, cell death determined by oligonucleosome release following transfection with *prepro-hIAPP* in the presence of 3-MA was significantly attenuated (Fig. 2b), which could be due to decreased accumulation of toxic hIAPP oligomer^17^ by MSL-7. Without 3-MA, cell death was not observed after transfection with *prepro-hIAPP* which is probably due to the effective clearance of hIAPP oligomer by constitutive autophagy (Fig. 2b), consistent with a previous report^3^.

To study effect of MSL-7 on IAPP oligomer clearance in a more physiological setting, we next studied whether MSL-7 can expedite clearance of endogenous IAPP oligomer. To this end, we employed monkey islet cells expressing amyloidogenic simian IAPP (sIAPP) that is almost identical to hIAPP^3,18^. When we studied whether MSL-7 induces the nuclear translocation of MiTF/TFE family members in monkey islet cells using confocal microscopy, markedly increased nuclear TFEB and TFE3 was observed after treatment with MSL-7 (Fig. 2c, d), similar to the results employing INS-1 cells. When we examined whether MSL-7 can induce autophagic activity in monkey islet cells using confocal microscopy upon transfection with *mRFP-GFP-LC3* tandem construct, the numbers of both autophagosomes and autophagolysosomes were significantly increased after treatment with MSL-7 (Fig. 2e), indicating that MSL-7 increases autophagic activity of monkey islet cells, similar to the results employing INS-1 cells. When primary monkey islet cells were cultured in the presence of 3-MA, sIAPP oligomer stained with A11 antibody recognizing hIAPP oligomer^19^ significantly accumulated which was not seen without 3-MA (Fig. 2f), suggesting that amyloidogenic sIAPP oligomer in monkey islet cells is constitutively cleared by autophagy. When monkey islet cells cultured in the presence of 3-MA were treated with MSL-7, accumulation of sIAPP oligomer was significantly reduced (Fig. 2f), suggesting that MSL-7 could expedite clearance of endogenous sIAPP oligomer. Furthermore, confocal microscopy after immunofluorescence revealed markedly increased colocalization of LC3 with A11^+^ sIAPP oligomer after treatment with MSL-7 in the presence of bafilomycin A1 inhibiting lysosomal degradation of sIAPP oligomer (Fig. 2g), supporting that sIAPP oligomer is cleared by autophagy. Apoptosis of monkey islet cells incubated with 3-MA probably due to accumulation of endogenous sIAPP oligomer^3^ was also significantly attenuated by MSL-7 (Fig. 2h), supporting that sIAPP oligomer is cleared by MSL-7-induced autophagy and that MSL-7-mediated reduction of sIAPP oligomer accumulation leads to the amelioration of cell death.

While behavior of sIAPP oligomer would be similar to that of hIAPP oligomer, we further investigated effect of MSL-7 on hIAPP oligomer clearance employing human β-cells or β-cell lines. We chose 1.1B4 cells that have been generated by electrofusion of human primary pancreatic islet cells to human pancreatic adenocarcinoma cells and express hIAPP^20^, instead of EndoC-βH1 cells that were derived from human fetal pancreas by SV40LT and human TERT transduction but virtually express no hIAPP^21^. MSL-7 treatment induced nuclear translocation of TFEB or TFE3, and enhanced formation of autophagosomes or autophagolysosomes in 1.1B4 cells (Supplementary Fig. 3a–c). MSL-7 reduced hIAPP oligomer accumulation and apoptosis in 1.1B4 cells incubated with 3-MA enhancing hIAPP oligomer accumulation (Supplementary Fig. 3d, e). MSL-7 induced expression of autophagy genes, lysosomal genes and *TFEB* or *TFE3* in 1.1B4 cells (Supplementary Fig. 3f and Supplementary Table 2), showing that MSL-7 effectively induced target genes of TFEB or TFE3, and enhanced clearance of hIAPP oligomer in human β-cells as well.

**Fig. 3:**
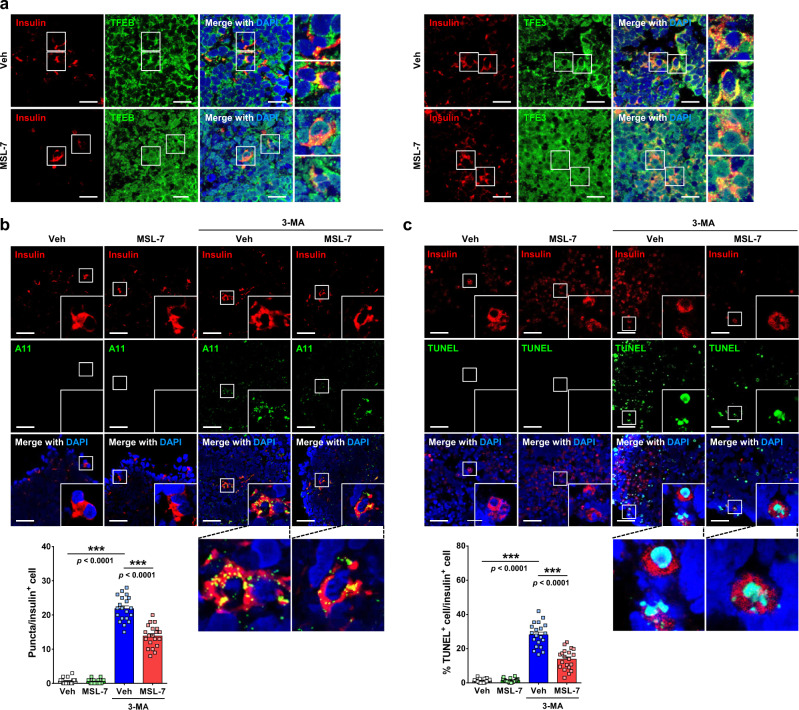
Effect of MSL-7 on the clearance of endogenous hIAPP oligomer in human β-cells differentiated from hiPSCs. **a** Expression of insulin and nuclear translocation of TFEB (left) or TFE3 (right) in human β-cells differentiated from human induced pluripotent stem cells (hiPSCs). Inset images were magnified to show cyan nuclei due to colocalization of TFEB with nuclear DAPI or yellow cytoplasm due to colocalization of TFEB with insulin^+^ cytoplasm. **b** Islet-like clusters differentiated from hiPSCs were treated with MSL-7 in the presence or absence of 3-MA for 16 h. After double immunofluorescence using A11 and anti-insulin antibodies as the primary antibodies, confocal microscopy was conducted and the number of A11^+^ puncta in insulin-producing β-cells was counted (*F* = 357.2, df treatment = 3, df residual = 76) (lower). Representative pictures are presented (upper). Inset images were magnified. **c** After treatment of islet-like clusters differentiated from hiPSCs cells with MSL-7 in the presence or absence of 3-MA for 16 h, combined TUNEL staining and insulin immunofluorescence were conducted, and the percentage of TUNEL^+^ apoptotic cells among insulin-producing β-cells was counted (*F* = 150.3, df treatment = 3, df residual = 76) (lower). Representative pictures are presented (upper). Inset images were magnified to show cyan nuclei due to colocalization of TUNEL staining with nuclear DAPI. All data in this figure are the means ± SEM from more than 3 independent experiments. (*n* = 20 islet-like clusters in **a**, **b**, **c**) (scale bar, 50 μm for **a** and 100 μm for **b**, **c**) ***P < 0.001 by one-way ANOVA with Tukey’s test.

Since behavior of 1.1B4 cells fused to pancreatic cancer cells could be different from normal human islet cells, we next used human islet cells differentiated from donor-derived human induced pluripotent stem cells (hiPSCs)^22^. hiPSCs were differentiated into β-cells using a kit (STEMdiff^TM^ Pancreatic Progenitor Kit, STEMCELL Technologies) according to a modification of previously reported methods^23,24^, which was confirmed by immunofluorescence using anti-insulin antibody (hiPSC-β-cells) (Fig. 3a and Supplementary Fig. 4a, b). The percentage of human insulin-producing cells among islet-like clusters was 7–8% (Supplementary Fig. 4b). When islet-like clusters differentiated from hiPSCs were incubated with 3-MA, hIAPP oligomer accumulation was clearly seen in insulin-producing cells as yellow puncta in double immunofluorescence using A11 and anti-insulin antibodies (Fig. 3b). Combined treatment with MSL-7 significantly reduced A11^+^ hIAPP oligomer accumulation in hiPSC-β-cells producing insulin (Fig. 3b). Reduced hIAPP oligomer accumulation was accompanied by nuclear translocation of TFEB and TFE3 as identified by immunofluorescence using specific antibodies, which can be seen as reduced cytosolic yellow fluorescence due to colocalization of insulin with TFEB or TFE3 and increased cyan fluorescence due to colocalization of nuclear DAPI with TFEB or TFE3 after MSL-7 treatment (Fig. 3a). When we determined apoptosis of hiPSC-β-cells in the presence of 3-MA cyan increasing A11^+^ hIAPP oligomer accumulation, percentage of TUNEL^+^ apoptotic cells (identified as cyan nuclei) among insulin^+^ cells was significantly reduced by MSL-7 (Fig. 3c), supporting that MSL-7 reduces human β-cell apoptosis by enhancing hIAPP oligomer clearance.

### Diminution of MSL-7 effects by *Tfeb*-knockout or *Tfe3*-knockout

**Fig. 4:**
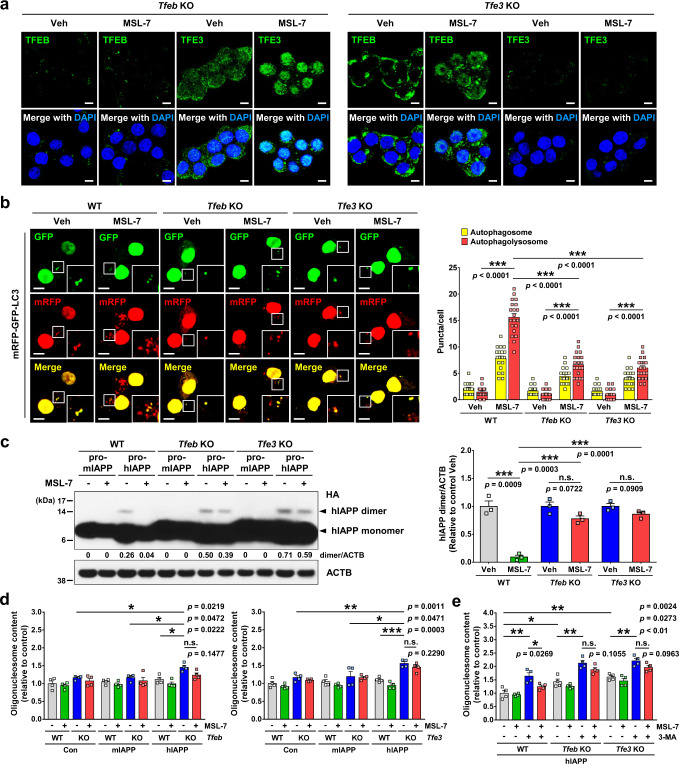
Reduced MSL-7 effects in *Tfeb-*KO or *Tfe3*-KO cells in vitro. **a** *Tfeb*-KO or *Tfe3*-KO INS-1 cells were treated with MSL-7 for 4 h, and immunostained using anti-TFEB (left) or anti-TFE3 antibody (right). After DAPI staining, cells were subjected to confocal microscopy. **b** *Tfeb*-KO or *Tfe3*-KO INS-1 cells transfected with *mRFP-GFP-LC3* tandem construct were treated with MSL-7 for 16 h, and the numbers of autophagosome and autophagolysosome were counted (*F* = 178.7, df treatment = 5, df residual = 114) (right). Representative pictures are presented (left). Inset images were magnified to show red (autophagolysosomes), green, and yellow (autophagosomes) puncta. **c** *Tfeb*-KO or *Tfe3*-KO INS-1 cells transfected with *prepro-mIAPP-HA* or *prepro-hIAPP-HA* were treated with MSL-7 for 16 h. Immunoblot analysis was conducted (*F* = 32.9, df treatment = 5, df residual = 12) (left). Densitometric value of HA bands normalized to the respective vehicle-treated cells (−) are presented (right). **d** *Tfeb*-KO (left) or *Tfe3*-KO (right) INS-1 cells transfected with *prepro-mIAPP-HA* or *prepro-hIAPP-HA* were treated with MSL-7 for 16 h without 3-MA, and apoptosis was measured (*F* = 5.8, df treatment = 11, df residual = 36 in left panel; *F* = 11.3, df treatment = 11, df residual = 36 in right panel). **e** *Tfeb*-KO or *Tfe3*-KO INS-1 cells transfected with *prepro-mIAPP-HA* or *prepro-hIAPP-HA* were treated with MSL-7 for 16 h in the presence of 3-MA, and apoptosis was measured (*F* = 24.1, df treatment = 11, df residual = 36). All data in this figure are the means ± SEM from more than 3 independent experiments performed in triplicate. (scale bar, 5 μm) *P < 0.05; **P < 0.01; ***P < 0.001 by one-way ANOVA with Tukey’s test. (n.s. not significant) Source data are provided as a Source Data file.

Since MSL-7 induces the nuclear translocation of TFEB and TFE3, critical regulators of lysosome biogenesis and autophagy gene expression, in pancreatic islet cells or INS-1 insulinoma cells, we next studied the role of MiTF/TFE family members in the accelerated clearance of hIAPP oligomer and improved viability of islet cells. To this end, we produced INS-1 cells with *Tfeb*-knockout or *Tfe3*-knockout (KO) using CRISPR/Cas9 technology. Immunoblot analysis demonstrated the absence of TFEB or TFE3 expression in *Tfeb-*KO or *Tfe3-*KO cells, respectively (Supplementary Fig. 5a). Nucleotide sequencing showed a deletion of 116 bp segment between the two sgRNA target loci of *Tfeb* and an insertion of 120 bp donor sequences to an sgRNA target locus of *Tfe3* (Supplementary Fig. 5b), which led to the decrease and increase of the band sizes of PCR product using flanking primer sequences, respectively (Supplementary Fig. 5c). Expression and nuclear translocation of TFE3 were not affected in *Tfeb*-KO cells, while those of TFEB were not affected in *Tfe3*-KO cells (Fig. 4a). When *Tfeb*-KO or *Tfe3*-KO INS-1 cells were transfected with *mRFP-GFP-LC3* plasmid and treated with MSL-7, the numbers of autophagosome and autophagolysosome were decreased in both *Tfeb*-KO and *Tfe3*-KO cells compared to control cells (Fig. 4b), suggesting reduced autophagic flux by *Tfeb*-KO or *Tfe3* KO. Furthermore, induction of autophagy genes and lysosomal genes by MSL-7 treatment was reduced in *Tfeb*-KO or *Tfe3*-KO INS-1 cells compared to control Cas9-treated cells, accounting for the decrease of MSL-7-induced autophagy in these cells (Supplementary Fig. 6a–i).

We also studied whether *Tfeb* or *Tfe3* KO affects hIAPP oligomer clearance by MSL-7. When cells were transfected with *prepro-hIAPP-HA*, intensity of hIAPP dimer bands before MSL-7 treatment was notably higher in both *Tfeb*-KO and *Tfe3*-KO INS-1 cells compared to control cells (compare 3rd with 7th or 11th lane from the left of Fig. 4c, left panel; Supplementary Fig. 1g, upper panel), probably because of reduced constitutive clearance of hIAPP dimer. When *Tfeb*-KO or *Tfe3*-KO INS-1 cells transfected with *prepro-hIAPP-HA* were treated with MSL-7, decreases of hIAPP dimer band intensity relative to vehicle (Veh)-treated *Tfeb*-KO or *Tfe3*-KO cells were small and statistically insignificant in both KO cells (compare 7th with 8th lane or 11th with 12th lane from the left of Fig. 4c, left panel; Supplementary Fig. 1g, upper panel). Thus, when normalized to the vehicle-treated respective KO cells, intensity of hIAPP dimer band after treatment of *Tfeb*-KO or *Tfe3*-KO INS-1 cells with MSL-7 was significantly higher than that after treatment of wild-type control cells with MSL-7 (compare 2nd with 4th or 6th bar from the left of Fig. 4c, right panel; Supplementary Fig. 1g, lower panel), suggesting diminished MSL-7 effect on hIAPP dimer clearance by *Tfeb* or *Tfe3* KO. Likely due to reduced constitutive clearance of hIAPP dimer, a small but significant apoptosis was observed after transfection of *Tfeb*-KO or *Tfe3*-KO INS-1 cells with *prepro-hIAPP* in the absence of 3-MA (compare 9th and 11th bars from the left of Fig. 4d, left and right panels), which was not observed in *Tfeb*-KO or *Tfe3*-KO cells transfected with *prepro-mIAPP* (compare 5th and 7th bars from the left of Fig. 4d, left and right panels) or autophagy-competent wild-type cells transfected with *prepro-hIAPP* (compare 1st and 9th bars from the left in the left and right panels of Fig. 4d). Apoptosis of *Tfeb*-KO or *Tfe3*-KO INS-1 cells after *prepro-hIAPP* transfection (in the absence of 3-MA) was not significantly reduced by MSL-7 (compare 11th and 12th bars from the left of Fig. 4d, left and right panels), likely due to decreases of MSL-7-induced autophagy in these cells. Apoptosis of *Tfeb*-KO or *Tfe3*-KO INS-1 cells after *prepro-hIAPP* transfection in the presence of 3-MA was also not significantly reduced by MSL-7 (compare 7th and 8th or 11th and 12th bars from the left of Fig. 4e), which is different from a significant decrease of *prepro-hIAPP*-transfected control INS-1 cell apoptosis in the presence of 3-MA by MSL-7 (compare 3rd and 4th bars from the left of Fig. 4e; see also Fig. 2b) and is probably due to reduced MSL-7-mediated autophagy in *Tfeb*-KO or *Tfe3*-KO INS-1 cells. Residual effect of MSL-7 in *Tfeb-*KO or *Tfe3*-KO INS-1 cells such as induction of low-level autophagy (Fig. 4b) and small increases of autophagy gene or lysosomal gene expression (Supplementary Fig. 6a–i) could be due to other members of MiTF/TFE family such as MiTF^25^ that is expressed in pancreatic islet cells at a low level (Supplementary Fig. 2a, b). Furthermore, TFE3 expression is intact in *Tfeb*-KO cells and TFEB expression is intact in *Tfe3*-KO cells (Supplementary Fig. 5a), which can explain residual induction of autophagy in *Tfeb*-KO or *Tfe3*-KO cells. However, autophagy induction in these cells was significantly reduced compared to wild-type cells, showing a significant role of *Tfeb* or *Tfe3* in autophagy induction by MSL-7.

### Effect of MSL-7 on transgenic mice expressing amyloidogenic hIAPP

**Fig. 5:**
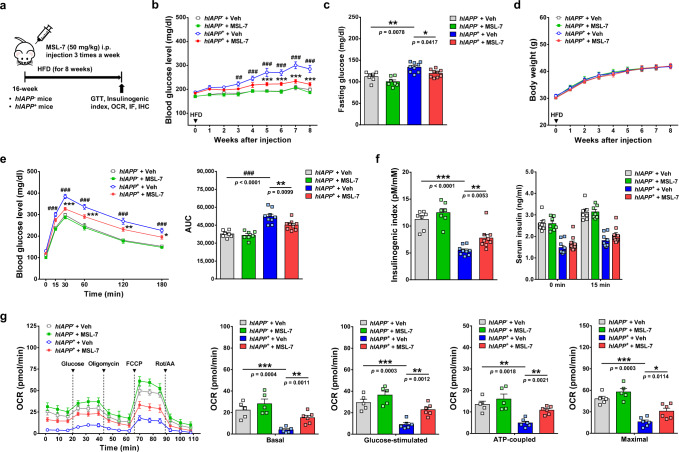
Metabolic effect of MSL-7 on *hIAPP*^+^ mice. **a** Scheme of MSL-7 treatment. **b** MSL-7 was administered intraperitoneally (i.p.) to HFD-fed male *hIAPP*^+^ mice. Nonfasting blood glucose was monitored (*F* = 35.0, df = 3). **c** Fasting blood glucose was determined in HFD-fed *hIAPP*^+^ mice treated with MSL-7 for 8 weeks (*F* = 14.3, df treatment = 3, df residual = 28). **d** Body weight of HFD-fed *hIAPP*^+^ mice was monitored during MSL-7 administration. **e** GTT was performed after MSL-7 administration to HFD-fed *hIAPP*^+^ mice (*F* = 24.4, df = 3) (left). AUC was calculated (*F* = 22.3, df treatment = 3, df residual = 28) (right). **f** Insulinogenic index was calculated after MSL-7 administration to HFD-fed *hIAPP*^+^ mice (*F* = 29.2, df treatment = 3, df residual = 28) (left). Serum insulin levels before and 15 min after glucose injection are shown (right). **g** Islets were isolated from HFD-fed *hIAPP*^+^ and *hIAPP*^−^ mice treated with vehicle (Veh) or MSL-7, and were subjected to respirometry using Seahorse XF analyzer (left). Basal (*F* = 13.7, df treatment = 3, df residual = 18), glucose-stimulated (*F* = 19.1, df treatment = 3, df residual = 18), ATP-coupled (*F* = 12.4, df treatment = 3, df residual = 18) and maximal O_2_ consumption (*F* = 23.7, df treatment = 3, df residual = 18) were calculated. All data in this figure are the means ± SEM from more than 3 independent experiments. (*n* = 7 for Veh-treated *hIAPP*^−^ mice, *n* = 7 for MSL-7-treated *hIAPP*^−^ mice, *n* = 9 for Veh-treated *hIAPP*^+^ mice and *n* = 9 for MSL-7-treated *hIAPP*^+^ mice in **b–f**; *n* = 5 for Veh-treated *hIAPP*^−^ mice, *n* = 5 for MSL-7-treated *hIAPP*^−^ mice treated, *n* = 6 for Veh-treated *hIAPP*^+^ mice and *n* = 6 for MSL-7-treated *hIAPP*^+^ mice in **g**) *P < 0.05; ^##^*P* or **P < 0.01; ^###^*P* or ***P < 0.001 by two-way ANOVA with Bonferroni’s test (**b, e**) and one-way ANOVA with Tukey’s test (**c, e, f, g**). (^#^, comparison between Veh-treated *hIAPP*^+^ and Veh-treated *hIAPP*^−^ mice; *, comparison between MSL-7-treated *hIAPP*^+^ and Veh-treated *hIAPP*^+^ mice in **b**, **e**).

Based on the in vitro results showing accelerated clearance of hIAPP oligomer by MSL-7, we next studied whether MSL-7 can improve the metabolic profile of transgenic mice expressing *hIAPP* in pancreatic β-cells (*hIAPP*^+^ mice) rendered diabetic by HFD. In vivo administration of 50 mg/kg MSL-7 3 times a week for 8 weeks (Fig. 5a) significantly decreased nonfasting and fasting glucose levels in *hIAPP*^+^ mice on HFD, while body weight was not significantly changed (Fig. 5b–d). Glucose tolerance test (GTT) showed that glucose intolerance in HFD-fed *hIAPP*^+^ mice was significantly ameliorated by MSL-7, accompanied by reduced area under the curve (AUC) (Fig. 5e). Insulinogenic index (the difference between serum insulin 0 and 15 min after glucose infusion divided by the difference between blood glucose 0 and 15 min after glucose infusion) representing β-cell function which was reduced in *hIAPP*^+^ mice on HFD (Fig. 5f), was also significantly improved by MSL-7 (Fig. 5f), suggesting that improved pancreatic β-cell function could explain amelioration of glucose intolerance of *hIAPP*^+^ mice on HFD by MSL-7 treatment. We also studied possible effect of MSL-7 on mitochondrial dysfunction caused by hIAPP oligomer^26,27^ which can affect glucose-induced insulin release^28^. Seahorse respirometry showed that basal, glucose-stimulated, ATP-coupled or maximal O_2_ consumption was suppressed in islets of *hIAPP*^+^ mice fed HFD (Fig. 5g), suggesting mitochondrial dysfunction of β-cells in these mice. Here, MSL-7 treatment in vivo ameliorated suppression of basal, glucose-stimulated, ATP-coupled or maximal O_2_ consumption of pancreatic islet cells of *hIAPP*^+^ mice on HFD, which could be due to increased turnover of dysfunctional mitochondria through autophagy activation and lead to improved β-cell function in these mice (Fig. 5g).

We did not observe improvement of metabolic indexes by MSL-7 administration to *hIAPP*^−^ mice on HFD for 8 weeks (Fig. 5a–f), which is different from significant metabolic improvement in C57BL/6 mice on HFD for 16 weeks by MSL-7 administration^9^. This discrepancy could be due to strain difference between C57BL/6 and FVB/N mice^29^ or duration of HFD feeding (8 vs. 16 weeks). Indeed, glucose tolerance determined by AUC of GTT curves was significantly impaired by HFD feeding of C57BL/6 mice for 8 weeks but not by that of FVB/N mice (Supplementary Fig. 7a). Furthermore, nonfasting blood glucose in FVB/N mice fed HFD for 8 weeks was not significantly elevated compared to that in the same mice fed normal chow diet (NCD) for 8 weeks (Supplementary Fig. 7b), suggesting that significant insulin resistance was not induced by HFD feeding of mice of FVB/N background and that effect of MSL-7 in *hIAPP*^+^ mice on HFD is due to its effect on β-cell function associated with hIAPP clearance but not due to improved insulin sensitivity. Consistently, we observed no effect of MSL-7 administration on insulin sensitivity of *hIAPP*^−^ or *hIAPP*^+^ mice on HFD as evidenced by no change of AUC of insulin tolerance test (ITT) curves by MSL-7 administration to *hIAPP*^−^ or *hIAPP*^+^ mice on HFD (Supplementary Fig. 7c), again indicating that metabolic improvement by MSL-7 administration to *hIAPP*^+^ mice is due to improved β-cell function but not due to improved insulin sensitivity. AUC of ITT curves was reduced in HFD-fed *hIAPP*^+^ mice compared to HFD-fed *hIAPP*^−^ mice both before (compare gray and blue curve or bar) and after (compare green and red curve or bar) MSL-7 administration showing reduced insulin sensitivity of HFD-fed *hIAPP*^+^ mice (Supplementary Fig. 7c), probably because elevated blood glucose level in HFD-fed *hIAPP*^+^ mice itself can reduce insulin sensitivity^30^.

**Fig. 6:**
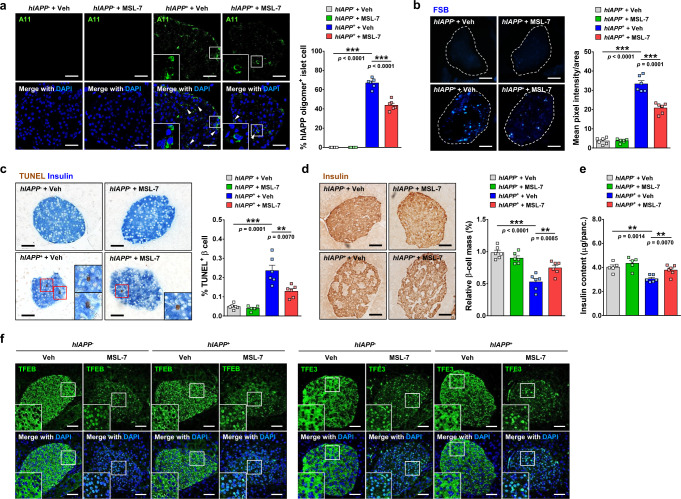
Pancreatic islets of *hIAPP*^+^ mice treated with MSL-7. **a** Percentage of A11 puncta^+^ cells among total DAPI^+^ islet cells in pancreatic sections from HFD-fed *hIAPP*^+^ mice treated with MSL-7 for 8 weeks was determined by confocal microscopy (*F* = 498.6, df treatment = 3, df residual = 20) (right). Representative pictures are shown (left). Inset images were magnified. Arrow heads indicate islet cells with pyknotic nuclei (*n* = 6 each, 132–148 islets per group). **b** Mean fluorescence intensity of FSB staining per islet area was determined using ImageJ (*F* = 178.3, df treatment = 3, df residual = 20) (right). Representative pictures are shown (left). (*n* = 6 each, 151–156 islets per group). **c** Percentage of TUNEL^+^ β-cells among total β-cells in sections of HFD-fed *hIAPP*^+^ mice treated with MSL-7 was calculated (*F* = 31.5, df treatment = 3, df residual = 20) (right). Representative TUNEL staining is shown (left). Insets (TUNEL^+^ β-cells) were magnified. (*n* = 6 each, 180–182 islets per group). **d** Relative β-cell mass in HFD-fed *hIAPP*^+^ mice treated with MSL-7 was determined by insulin immunohistochemistry and point counting (*F* = 22.2, df treatment = 3, df residual = 20) (right). Representative insulin immunochemistry is shown (left). (*n* = 6 each, 180–182 islets per group). **e** Insulin content of pancreatic islets from HFD-fed *hIAPP*^+^ and *hIAPP*^−^ mice treated with MSL-7 was measured by ELISA, which was normalized to the total pancreas weight (*F* = 9.5, df treatment = 3, df residual = 18). (*n* = 5 for Veh-treated *hIAPP*^−^ mice, *n* = 5 for MSL-7-treated *hIAPP*^−^ mice, *n* = 6 for Veh-treated *hIAPP*^+^ mice and *n* = 6 for MSL-7-treated *hIAPP*^+^ mice). **f** Immunfluorescence of pancreatic sections from HFD-fed *hIAPP*^+^ and *hIAPP*^−^ mice treated with MSL-7 using anti-TFEB (left) and anti-TFE3 antibodies (right). Insets were magnified to show colocalization of TFEB/TFE3 with nuclear DAPI. (*n* = 6 each) All data in this figure are the means ± SEM from more than 3 independent experiments. (scale bar, 100 μm) **P < 0.01; ***P < 0.001 by one-way ANOVA with Tukey’s test.

To investigate whether improved glucose profile and β-cell function in vivo by MSL-7 administration were due to the increased clearance of toxic hIAPP oligomer, we conducted immunofluorescence of pancreatic islets using A11 antibody. A11^+^ hIAPP oligomer accumulation in pancreatic islets of *hIAPP*^+^ mice on HFD was significantly reduced by in vivo administration of MSL-7 for 8 weeks (Fig. 6a). Some islet cells with A11^+^ hIAPP oligomer had pyknotic nuclei, suggesting occurrence of apoptosis by hIAPP oligomer accumulation (Fig. 6a). Amyloid staining using (*E*,*E)*-1-fluoro-2,5-bis(3-hydroxycarbonyl-4-hydroxy) styrylbenzene (FSB) showed that pancreatic islet amyloid accumulation observed in *hIAPP*^+^ mice on HFD was also significantly reduced by in vivo administration of MSL-7 for 8 weeks (Fig. 6b). The number of TUNEL^+^ apoptotic β-cells, which was increased in HFD-fed *hIAPP*^+^ mice, was significantly reduced by MSL-7 treatment for 8 weeks as well (Fig. 6c). Accordingly, β-cell mass determined after insulin immunohistochemistry which was reduced in *hIAPP*^+^ mice on HFD was significantly recovered by MSL-7 treatment for 8 weeks (Fig. 6d). Insulin content of the pancreas which was reduced in *hIAPP*^+^ mice on HFD was also restored by MSL-7 treatment for 8 weeks (Fig. 6e). When we studied whether MSL-7 administration could change the distribution of MiTF/TFE family members in pancreatic islets in vivo, nuclear translocation of TFEB and TFE3 in islet cells was clearly observed after 8 weeks of MSL-7 administration as evidenced by cyan nuclei due to colocalization of TFEB and TFE3 fluorescence with nuclear DAPI (Fig. 6f), suggesting that increased activity of MiTF/TFE family members leading to increased autophagic activity is responsible for the increased clearance of islet hIAPP oligomer in vivo and improved β-cell function.

We also studied whether MSL-7 could enhance in vivo clearance of hIAPP oligomer that accumulates through mechanisms other than HFD feeding of *hIAPP*^+^ mice. When we administered MSL-7 to *hIAPP*^+^ mice treated with a low dose of streptozotocin (STZ) that has been reported to induce accumulation of hIAPP amyloid without causing extensive β-cell destruction^5^, contents of hIAPP oligomer and islet amyloid in pancreatic islets of STZ-treated *hIAPP*^+^ mice were significantly reduced (Supplementary Fig. 8a, b), suggesting that MSL-7 is able to expedite clearance of hIAPP oligomer and amyloid accumulating through diverse pathways. Consistently, elevated nonfasting blood glucose and glucose intolerance after STZ administration to *hIAPP*^+^ mice were significantly reduced by MSL-7 treatment in vivo (Supplementary Fig. 8c, d).

To finally confirm that effect of MSL-7 improving metabolic profile of *hIAPP*^+^ mice on HFD is due to enhanced autophagy or TFEB activation in pancreatic β-cells, we generated *hIAPP*^+^ mice with β-cell-specific *Tfeb*-KO (*hIAPP*^+^*Tfeb*^Δβ-cell^) by breeding *hIAPP*^+^ mice with RIP-*Cre* × *Tfeb*^F/F^ mice. Nonfasting or fasting blood glucose level after HFD feeding was further increased in *hIAPP*^+^*Tfeb*^Δβ-cell^ mice compared to HFD-fed control *hIAPP*^+^*Tfeb*^F/F^ mice probably due to reduced autophagy or lysosomal function in β-cells, while body weighs were not different between groups (Supplementary Fig. 9a–c). HFD-induced impairment of glucose tolerance measured by AUC of GTT curves and HFD-induced decrease of insulinogenic index in control *hIAPP*^+^*Tfeb*^F/F^ mice were also aggravated in *hIAPP*^+^*Tfeb*^Δβ-cell^ mice (Supplementary Fig. 9d, e), again probably due to reduced constitutive autophagy or lysosomal function by β-cell-specific *Tfeb*-KO.

**Fig. 7:**
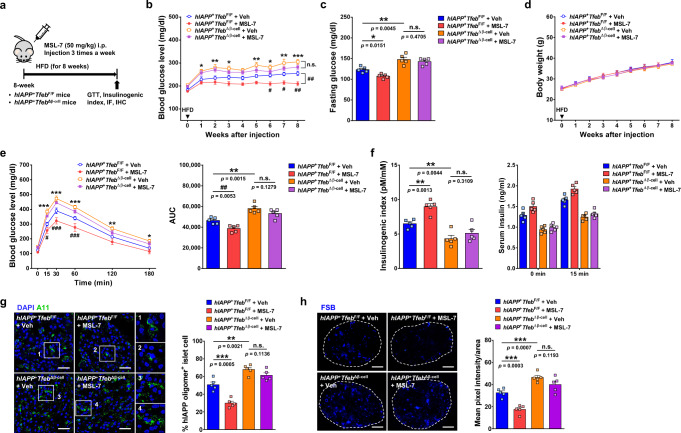
Role of pancreatic β-cell *Tfeb* in MSL-7-induced metabolic improvement. **a** Scheme of MSL-7 administration to *hIAPP*^+^*Tfeb*^Δβ-cell^ mice. **b** Nonfasting blood glucose was monitored weekly during MSL-7 treatment (*F* = 28.0, df = 3). (*n* = 5 each). **c** Fasting blood glucose in HFD-fed *hIAPP*^+^*Tfeb*^Δβ-cell^ and *hIAPP*^+^*Tfeb*^F/F^ mice treated with MSL-7 (*F* = 23.2, df treatment = 3, df residual = 16). (*n* = 5 each). **d** Body weight before and during MSL-7 treatment of HFD-fed *hIAPP*^+^*Tfeb*^Δβ-cell^ and *hIAPP*^+^*Tfeb*^F/F^ mice. (*n* = 5 each). **e** GTT in HFD-fed *hIAPP*^+^*Tfeb*^Δβ-cell^ or *hIAPP*^+^*Tfeb*^F/F^ mice treated with MSL-7 (*F* = 26.9, df = 3) (left). AUC was calculated (*F* = 23.4, df treatment = 3, df residual = 16) (right). (*n* = 5 each). **f** Insulinogenic index in HFD-fed *hIAPP*^+^*Tfeb*^Δβ-cell^ or *hIAPP*^+^*Tfeb*^F/F^ mice treated with MSL-7 (*F* = 21.0, df treatment = 3, df residual = 16) (left). Serum insulin before and 15 min after glucose injection (right). (*n* = 5 each). **g** Percentage of A11^+^ cells among total islet cells in sections of HFD-fed *hIAPP*^+^*Tfeb*^Δβ-cell^ or *hIAPP*^+^*Tfeb*^F/F^ mice treated with MSL-7 (*F* = 40.9, df treatment = 3, df residual = 16) (right). Representative pictures were presented (left). Inset images were magnified. (*n* = 5 each, 36–45 islets). **h** Mean pixel intensity of FSB per islet area in sections from HFD-fed *hIAPP*^+^*Tfeb*^Δβ-cell^ or *hIAPP*^+^*Tfeb*^F/F^ mice treated with MSL-7 (*F* = 32.0, df treatment = 3, df residual = 16) (right). Representative pictures were presented (left). (*n* = 5 each, 40–51 islets). All data in this figure are the means ± SEM from more than 3 independent experiments. (scale bar, 100 μm) ^#^*P* or *P < 0.05; ^##^*P* or **P < 0.01; ^###^*P* or ***P < 0.001 by two-way ANOVA with Bonferroni’s test (**b, e**) and one-way ANOVA with Tukey’s test (**c, e, f, g, h**). (^#^, comparison between MSL-7-treated *hIAPP*^+^*Tfeb*^F/F^ and Veh-treated *hIAPP*^+^*Tfeb*^F/F^ mice; *, comparison between Veh-treated *hIAPP*^+^*Tfeb*^Δβ-cell^ and Veh-treated *hIAPP*^+^*Tfeb*^F/F^ mice in **b**, **e**). (n.s. not significant).

In these mice, we studied whether in vivo effects of MSL-7 is affected by *Tfeb* of β-cells. We started MSL-7 treatment of *hIAPP*^+^*Tfeb*^Δβ-cell^ mice at the age of 8 weeks (Fig. 7a) because nonfasting blood glucose began to rise in 8-week-old *hIAPP*^+^*Tfeb*^Δβ-cell^ mice on NCD (see Supplementary Fig. 9a), and it might be difficult to compare MSL-7 effect between *hIAPP*^+^*Tfeb*^Δβ-cell^ and *hIAPP*^+^*Tfeb*^F/F^ mice on HFD if basal nonfasting glucose levels before MSL-7 treatment were significantly different between these mice. In *hIAPP*^+^*Tfeb*^Δβ-cell^ mice on HFD, reduction of nonfasting or fasting blood glucose by MSL-7 treatment (differences between orange and violet lines or bars in Fig. 7b, c) was statistically insignificant and less compared to that in *hIAPP*^+^*Tfeb*^F/F^ mice on HFD (differences between blue and red lines or bars in Fig. 7b, c) without differences of body weight between groups (Fig. 7d), supporting that enhanced TFEB activation or autophagy contributes to the improved glucose profile of MSL-7-treated *hIAPP*^+^ mice on HFD. Improvement of glucose tolerance measured by AUC of GTT curves or that of insulinogenic index after MSL-7 administration to *hIAPP*^+^*Tfeb*^Δβ-cell^ mice on HFD (differences between orange and violet lines or bars in Fig. 7e, f) was also statistically insignificant and less compared to that in *hIAPP*^+^*Tfeb*^F/F^ mice on HFD (differences between blue and red lines or bars in Fig. 7e, f), suggesting the role of enhanced TFEB activation or autophagy in the improvement of glucose tolerance and β-cell function by MSL-7 administration to *hIAPP*^+^ mice on HFD. Consistently, reduction of hIAPP oligomer or FSB-stained islet amyloid accumulation after MSL-7 administration to *hIAPP*^+^*Tfeb*^Δβ-cell^ mice on HFD (differences between orange and violet bars in Fig. 7g, h) was statistically insignificant and less compared to that in *hIAPP*^+^*Tfeb*^F/F^ mice on HFD (differences between blue and red bars in Fig. 7g, h).

## Discussion

We here have demonstrated that the autophagy enhancer small molecule, MSL-7 that could improve metabolic profile of obese mice by activating TFEB and enhancing lysosomal biogenesis or autophagy gene expression, can also improve glucose profile and pancreatic β-cell function of transgenic mice expressing amyloidogenic *hIAPP* fed HFD. Improvement of pancreatic β-cell function in vivo can be explained by expedited clearance of hIAPP oligomer and amelioration of pancreatic β-cell death caused by hIAPP oligomer accumulation. MSL-7-induced reduction of amyloidogenic IAPP oligomer accumulation and resultant cell death in the presence of 3-MA was observed not only when hIAPP was overexpressed by genetic methods but also when primary monkey islet cells expressing endogenous amyloidogenic sIAPP or hiPSC-derived β-cells expressing hIAPP were employed, suggesting physiological relevance of MSL-7 effect improving pancreatic β-cell viability. In addition to the reduction of hIAPP oligomer-mediated β-cell death, ameliorated mitochondrial dysfunction by MSL-7 may also contribute to the improved β-cell function of HFD-fed *hIAPP*^+^ mice because hIAPP oligomer can cause mitochondrial damage and mitochondrial function is crucial in the insulin release in response to glucose^27,28^. While we observed similar cell death following inhibition of autophagy in both genetic overexpression of hIAPP and endogenous amyloidogenic hIAPP models, accumulated hIAPP species were different between them because pro-hIAPP dimer or trimer was observed in INS-1 cells transfected with *prepro-hIAPP-HA*, while hIAPP oligomer stained with A11 antibody was observed in hiPSC-derived β-cells expressing endogenous amyloidogenic hIAPP. We have no direct biochemical evidence showing the progression from pro-hIAPP dimer to high-*n* hIAPP oligomer or amyloid. However, we have observed the proportional relationship between pro-hIAPP dimer and hIAPP oligomer in the same cells – monkey islet cells transfected with *prepro-hIAPP-HA*^3^, and it is likely that (pro-)hIAPP dimer or trimer acts as a seed for high-*n* hIAPP oligomer^16^. Our study also suggests the possibility that while pro-hIAPP dimer may exist in unstressed β-cells and is not cytotoxic, further progression into high-*n* oligomer occurs in the presence of autophagy impairment or metabolic stress^3^. It should also be noted that A11 antibody that was raised against human Aβ oligomer and has been used to detect hIAPP oligomer, can recognize other proteins such as heat-shock proteins^31^.

Metabolic effect of MSL-7 appears to be due to the activation of the MiTF/TFE family members enhancing autophagic activity and lysosomal function. In our CRISPR/Cas9 KO experiments, both *Tfeb* and *Tfe3* appear to contribute to the expedited clearance of hIAPP oligomer and reduced cell death by MSL-7 because the reduction of hIAPP dimer accumulation and hIAPP-associated cell death by MSL-7 became statistically insignificant by KO of either *Tfeb* or *Tfe3*, suggesting additive effects of *Tfeb* and *Tfe3* in the protection of insulinoma cell death by MSL-7. Consistently, dephosphorylation and nuclear translocation by MSL-7 occurred in both TFEB and TFE3 proteins, which could be dependent on calcineurin^9^. Nuclear translocation of both TFEB and TFE3 by MSL-7 was observed not only in vitro but also in vivo, which appears to be responsible for reduced hIAPP oligomer/amyloid accumulation in pancreatic islets, ameliorated pancreatic β-cell death, enhanced the pancreatic β-cell function and improved the glucose tolerance of HFD-fed *hIAPP*^+^ mice. Direct evidence supporting the role of pancreatic β-cell TFEB in the clearance of hIAPP oligomer was obtained using β-cell-specific *Tfeb*-KO mice crossed to *hIAPP*^+^ mice. In those *hIAPP*^+^*Tfeb*^Δβ-cell^ mice on HFD, blood glucose profile, glucose tolerance and insulinogenic index were further deteriorated compared to control *hIAPP*^+^*Tfeb*^F/F^ mice on HFD. Moreover, improvement of these metabolic parameters by MSL-7 administration was also diminished in *hIAPP*^+^*Tfeb*^Δβ-cell^ mice on HFD, indicating in vivo role of β-cell TFEB in MSL-7-mediated clearance of hIAPP oligomer.

We have previously reported that autophagy enhancers such as MSL-7 could be a therapeutic agent against diabetes or metabolic syndrome associated with obesity based on our data using mouse models. However, human diabetes is different from murine diabetes in that islet amyloid is found in most patients with type 2 diabetes due to the amyloidogenic propensity of hIAPP^2,32^. Since the aggregate-prone or amyloid proteins are cleared preferentially by autophagy rather than by the proteasomal degradation pathway^6^, the role of autophagy or the effect of autophagy modulators may be more important in human type 2 diabetes than in murine type 2 diabetes, which was strengthened by our observation that MSL-7 can expedite clearance of hIAPP oligomer accumulation in hiPSC-β-cells and protect those cells from hIAPP oligomer-induced cell death. MSL-7 or other autophagy enhancers could be therapeutic agents against human diabetes associated with islet amyloid accumulation.

## Methods

### Animals

*hIAPP*^+^ mice expressing *hIAPP* under the control of the rat insulin II promoter (FVB/N-Tg (Ins2-IAPP) RHFSoel/J mice, Stock No. 008232, Jackson Laboratory) were maintained on an FVB/N background, and genotyping was conducted by PCR analysis of tail DNA using specific primers (forward, 5’-GTCATGTGCACCTAAAGGGGCAAGTAATTCA-3’; reverse, 5’-CGAGTGGGCTATGGGTTTGT-3’)^3^. *Tfeb*^F/F^ mice were generated by breeding *Tfeb*^tm1a(EUCOMM)Wtsi^ mice (Mutant Mouse Resource and Research Center, MMRRC) with FLPeR mice (Wellcome Trust Sanger Institute). *hIAPP*^+^ mice with targeted disruption of *Tfeb* in pancreatic β-cells were generated by crossing RIP-*Cre* mice (Jackson Laboratory) with *Tfeb*^F/F^ mice and then with *hIAPP*^+^ mice (*hIAPP*^+^*Tfeb*^Δβ-cell^ mice). GTT was conducted by the intraperitoneal injection of 1 g/kg glucose to overnight fasted mice. Blood glucose concentration was measured using a One Touch glucometer (Lifescan) at 0, 15, 30, 60, 120, and 180 min after glucose injection^3^. ITT was performed by intraperitoneal injection of 1 U/kg regular insulin to fasted mice and measuring blood glucose levels at 0, 15, 30, 60, 120, and 180 min after insulin injection^33^. Serum insulin levels before and 15 min after the glucose challenge were measured using an ELISA kit (Shibayagi Co.) to calculate insulinogenic index (∆insulin_15min_/∆glucose_15min_)^3^.

Cynomolgus monkeys purchased from Guangxi Grandforest Scientific Primate Company Ltd. were maintained in the Orientbio Animal Facility^3^.

All animals were maintained in a specific pathogen free (SPF) facility accredited by the Association for the Assessment and Accreditation of Laboratory Animal Care International (AAALAC). All animal experiments were approved by the Institutional Animal Care and Use Committee of Yonsei University Health System (IACUC of YUHS) and were conducted in accordance with the Public Health Service Policy on Humane Care and Use of Laboratory Animals. Monkey experiments were approved by the IACUC of Orientbio, another AAALAC-accredited unit.

MSL-7 was dissolved in DMSO to make 125 mg/ml stock solution, and diluted with PBS to 5 mg/ml for injection (50 mg/kg, intraperitoneally 3 times a week for 8 weeks). To study MSL-7 effect on STZ-induced hIAPP oligomer accumulation, STZ (Sigma) dissolved in 0.1 M citrate buffer, pH 4.5, was injected into mice intraperitoneally to 2-month-old male *hIAPP*^+^ mice at a dose of 80 mg/kg. Twenty-four h later, MSL-7 administration was started and continued every other day for a total of 3 times.

### Isolation of pancreatic islets

Primary monkey islet cells were isolated using the modified Ricordi’s method^34^ as previously described^3^. In brief, the pancreas dissected from a 60-month-old male monkey was placed in 4 °C HTK solution for distention with 3–4 ml/g pancreas cold Liberase MTF C/T solution (Roche Custom Biotech). The distended pancreas was placed in an isolation chamber equipped with a peristaltic pump, and the temperature was raised to 37 °C. The isolation chamber was gently shaken during digestion, and serial samples were examined under a light microscope after dithizone (Sigma Aldrich) staining. When liberated pancreatic islets were observed, Dilution Solution (Corning) was added to inactivate Liberase MTF C/T. Digested cells collected into 200 ml conical tubes were washed with Washing Solution (Corning). Islets were then isolated by continuous density gradient centrifugation using a COBE 2991 Cell Processor (Terumo BCT Inc.) in iodixanol density gradient media (Optiprep^TM^ Axis-Shield, Alere Technologies AS) for culture in CMRL1066-10% FCS at 37 °C.

Primary murine islets were isolated from fasted mice using the collagenase digestion technique as previously described^35^. In brief, after injection of 2.5 ml of collagenase P (0.8 mg/ml) into the common bile duct, the pancreas was procured and incubated in a collagenase solution at 37 °C for 13 min 20 s. After cessation of enzymatic digestion with cold HBSS-5% FCS, tissue was passed through a 400 μm sieve and then centrifuged on 1.10, 1.085, 1.069 and 1.037 g/ml Biocoll gradients (Biochrom). Islets were collected from the interface using micropipettes.

### Cells

INS-1 cells (kindly provided by Dr. C. Wollheim, University of Geneva) were cultured in RPMI-1640 supplemented with 10% FCS, 1 mM sodium pyruvate (Sigma-Aldrich), 10 mM HEPES (Corning), 50 μM 2-mercaptoethanol (Sigma-Aldrich) and 100 U/ml penicillin-100 μg/ml streptomycin (Lonza) (passage number, 89). 1.1B4 cells were obtained from ECACC (Salisbury) through Fadzilah Adibah Abdul Majid (Universiti MalaysiaTerengganu), and cultured in RPMI 1640-10%FCS-100 U/ml penicillin-100 μg/ml streptomycin (passage number, 35). MSL-7 was dissolved in DMSO to obtain 20 mM stock solution and directly diluted to the final concentrations in culture medium for in vitro experiments. All cells were free of mycoplasma contamination. All in vitro experiments were repeated at least three times to confirm reproducibility.

### hiPSC generation and differentiation into insulin-producing cells

hiPSCs were derived by umbilical cord blood mononuclear cells, and reprogramming was conducted using CytoTune-iPS Sendai Reprogramming Kit (Life Technologies). Healthy human cord blood had been obtained from newborn after obtaining informed parental consent at Catholic Hematopoietic Stem Cell Bank of Korea. hiPSCs were adapted to feeder-free conditions on vitronectin (VTN-N) (Gibco)-coated plates in TeSR^TM^-E8^TM^ medium (STEMCELL Technologies). These cells were incubated at 37 °C in a 5% CO_2_ atmosphere, and the medium was changed daily. To initiate differentiation, confluent hiPSCs were dissociated into single cell suspension using TrypLE and seeded at 1.0 × 10^6^ cells per well in Aggrewell^TM^ 400 plates (STEMCELL Technologies) for incubation in AggreWell^TM^ medium with 10 μM Rho kinase inhibitor (Y-27632). After 24 h of incubation, aggregated cells were differentiated into insulin-producing cells using STEMdiff^TM^ Pancreatic Progenitor Kit (STEMCELL Technologies). At the stage for pancreatic progenitor generation, 0.25 μM SANT1 (Sigma-Aldrich) was added for the last 2 days. Cells were then cultured in CMRL medium (Welgene) containing 25 ng/ml human HGF (PeproTech), 10 mM nicotinamide (Sigma-Aldrich), 20 ng/ml Exendin-4 (ProSpec), 1 μM T3 (Sigma-Aldrich) and 10 μM Alk5 inhibitor (Biogems) for 6 days to produce insulin-producing cells. For immunofluorescence or TUNEL staining of iPSC-β-cells, islet-like clusters were pre-embedded in agar before paraffin embedding. All experiments using hiPSC were conducted in accordance with the protocol approved by the IRB of the Catholic University of Korea.

### TUNEL staining and β-cell mass

Deparaffinized pancreatic sections were incubated with TUNEL reagent (Roche Applied Science) and DAB (Life Technologies) as the color substrate. Insulin immunochemistry was then conducted by serial incubation with anti-insulin antibody (Cell Signaling Technology, 1:150), biotinylated anti-rabbit antibody (Vector Laboratories, 1:100), streptavidin-alkaline phosphatase, and then with Vector® Blue alkaline phosphatase substrate^3^ (Vector Laboratories). The percentage of TUNEL^+^ cells among total β-cells was determined in more than 30 islets per mouse (more than 180 islets per group) by manual counting under BX43 microscope (Olympus). To detect TUNEL^+^ cells among hiPSC-β-cells, TUNEL staining was followed by incubation with anti-insulin antibody and then with Alexa 594-anti-mouse IgG (Life Technologies, 1:200) which was subjected to fluorescent confocal microscopy using LSM700 microscope (Carl Zeiss). The percentage of TUNEL^+^ cells among insulin^+^ cells was determined in more than 20 islet-like clusters by manual counting. Relative β-cell mass was determined by analyzing more than 30 islets per mouse (more than 180 islets per group). After insulin immunohistochemistry using anti-insulin antibody and DAB, point counting morphometry was conducted as previously described^3^.

### IAPP expression in vitro

The transient transfection of *prepro-hIAPP-HA* and *prepro-mIAPP*-*HA*^3^ was conducted using jetPEI® DNA transfection reagent (Polyplus Transfection)^3^. To block autophagy of transfected cells, after 24 h of transfection, the culture medium was changed to a new medium containing 5 mM 3-MA (Sigma Aldrich) or 10 nM bafilomycin A1 (Sigma Aldrich). After another 16 h of incubation with or without MSL-7, cells were subjected to electrophoretic separation after lysis, immunofluorescence or oligonucleosome detection by ELISA^3^.

### Generation of CRISPR/Cas9 KO cells

To make *Tfeb*-KO or *Tfe3*-KO INS-1 cells, cells were transfected with 2 μg each of sgRNA plasmid (*Tfeb* sgRNA1, CATGCAGCTCATGCGGGAGCAGG; *Tfeb* sgRNA2, TGAACTGGGGTGTTGATGGCTGG; *Tfe3* sgRNA1, TGCTGCGGCAGCAGCTTATGAGG; *Tfe3* sgRNA2, GGGGTGGACGACTCAATGTGTGG), and *Cas9-Puro2A-RFP* using jetPEI® DNA transfection reagent (Polyplus Transfection). After puromycin selection, genomic DNA was isolated, followed by two rounds of PCR using specific primers (*Tfeb* 1st-F, AGGGCAGGAACAGGATGATG; *Tfeb* 1st-R, CCAGCAGAGTTGCAAGACGA; *Tfeb* 2nd-F, GATGTGGATGTGACAGCGAG; *Tfeb* 2nd-R, GACTGTTGGGGGCACTGTTG; *Tfe3* 1st-F, GCTCCAGCGTAGGTTTAGCA; *Tfe3* 1st-R, CACGAGGACCCTGAGTGATG; *Tfe3* 2nd-F, TAGCTTACCTGTGGCCCTGT; *Tfe3* 2nd-R, GGACCCTGAGTGATGATTCCT) and a T7E1 cleavage assay using a kit (New England Biolabs). Cells with mismatched mutations were isolated by the low-density seeding method. Gene KO was confirmed by nucleotide sequencing using an automatic DNA analyzer (Bioneer).

### Confocal microscopy

Immunofluorescence of MiTF/TFE family members in INS-1 cells, 1.1B4 cells or primary pancreatic islet cells was conducted using anti-TFEB (Bethyl Laboratories, 1:150), anti-TFE3 (Sigma Aldrich, 1:150), or anti-MiTF (Thermo Fisher Scientific, 1:150) as the primary antibodies. For TFEB immunostaining of INS-1, 1.1B4 or primary islet cells, cells with nuclear TFEB were considered to be activated even though residual cytosolic TFEB is detected because TFEB staining was observed only in the cytoplasm and nuclear TFEB was almost completely absent in most of untreated cells, while nuclear TFEB staining with residual cytosolic TFEB staining was seen after treatment with MSL-7. For TFE3 immunostaining, cells with bright nuclear TFE3 without cytosolic TFE3 were considered to be activated because faint nuclear TFE3 was observed in untreated cells, while bright nuclear TFE3 staining without cytosolic TFE3 staining was observed in most cells after MSL-7 treatment. The percentage of cells with nuclear TFEB or TFE3 among total DAPI^+^ cells was determined by counting more than 100 cells from more than 5 fields manually.

hIAPP oligomer in frozen tissue sections or in cells cultured in a Lab-Tak® II Chamber Slide (Thermo Fisher Scientific) was identified by immunostaining using A11 antibody (Merck Millipore, 1:150) and Alexa 488-anti-rabbit IgG (Life Technologies, 1:200). To analyze IAPP oligomer accumulation in cultured cells, confocal microscopy was conducted using LSM700 microscope, and the numbers of A11^+^ puncta in more than 20 cells were counted manually. To study hIAPP oligomer accumulation in the pancreas, the percentage of A11^+^ cells among total DAPI^+^ islet cells in pancreatic sections was determined by manually counting more than 20 islets per mouse (more than 132 islets per group). To investigate colocalization of A11 puncta with autophagosome, immunostaining using A11 antibody was followed by immunostaining using anti-LC3 antibody (MBL, 1:150) and then with Alexa 594-anti-mouse IgG (Life Technologies, 1:200). Confocal microscopy was conducted using LSM700 microscope, and the number of yellow puncta (autophagosome colocalized with A11) was counted manually in more than 20 cells.

### Amyloid staining

FSB staining for tissue amyloid was conducted using a modification of previously reported methods^36,37^. Briefly, pancreatic sections were deparaffinized in xylene and hydrated down to 30% ethanol. Deparaffinized sections were incubated in 70% formic acid for 20 min. After incubation with 10 μM FSB (Merck Millipore) for 1 h at room temperature with light protection, sections were subjected to fluorescent microscopy using BX53F fluorescent microscope (Olympus). Mean pixel intensity per islet area was determined in more than 25 islets per mouse (more than 151 islets per group) using ImageJ software (NIH).

### Transfection and plasmids

Cells were transiently transfected with plasmids such as *Tfeb-GFP or Tfe3-GFP* using jetPEI® DNA transfection reagent. Nuclear translocation of *Tfeb-GFP or Tfe3-GFP* was determined after treatment of transfected INS-1, 1.1B4 or primary monkey islet cells with MSL-7 for 4 h by confocal microscopy. To examine autophagosomes and autophagolysosomes, tandem *mRFP*-*GFP*-*LC3* was transfected using lipofectamine 2000 (Life Technologies). Confocal microscopy was conducted after treatment of transfected cells with MSL-7 for 4 h using LSM700 microscope. GFP or RFP puncta were defined as non-nuclear punctate structures with discrete fluorescent signal on a dark background which was clearly visible with fluorescent confocal microscopy. The numbers of yellow puncta (autophagosomes) and red puncta (autophagolysosomes) in more than 20 cells were counted manually.

### Immunoblot analysis and antibodies

Cells were lysed with a buffer (1% SDS, 100 mM Tris, pH 7.5, 0.5% Triton X-100) containing protease and phosphatase inhibitors. Protein concentration was determined using the Bradford method. Samples (5–10 μg) were separated on 4–12% Bis-Tris gel (NuPAGE®, Life Technologies), and transferred to nitrocellulose membranes (Merk Millipore) for immunoblot analysis using the enhanced chemiluminescence (ECL) method (Dongin LS). Antibodies against the following proteins were used: SQSTM1 (Progen Biotechnik, 1:5000), LC3 (Novus Biologicals, 1:1000), TFEB (Bethyl Laboratories, 1:1000), TFE3 (Sigma Aldrich, 1:1000), phospho-S142-TFEB (Merk Millipore, 1:1000), phospho-(Ser) 14-3-3 binding motif (Cell Signaling Technology, 1:1000), 14-3-3 protein (Cell Signaling Technology, 1:1000), β-actin (ACTB) (Santa Cruz Biotechnology, 1:4000), HA (Cell Signaling Technology, 1:1000) or Lamin A (Santa Cruz Biotechnology, 1:1000). Densitometry of the protein bands was performed using ImageJ.

### Immunoprecipitation

After lysis of *Tfeb*-*GFP* or *Tfe3-GFP* transfectant or nontransfectant INS-1 cells treated with MSL-7 for 4 h in ice-cold lysis buffer (400 mM NaCl, 25 mM Tris–HCl, pH 7.4, 1 mM EDTA, and 1% Triton X-100) containing protease and phosphatase inhibitors, lysates were centrifuged at 13,000 × *g* for 10 min in a microfuge tube and incubated with anti-GFP (1:1000, AbFrontier), anti-TFEB (Bethyl Laboratories, 1:1000) or anti-TFE3 antibody (Sigma Aldrich, 1:1000) in binding buffer (200 mM NaCl, 25 mM Tris–HCl, pH 7.4, 1 mM EDTA) with constant rotation at 4 °C for 1 h. After adding 50 μl of 50% of Protein-G beads (Roche Applied Science) slurry to the lysates and incubation with rotation at 4 °C overnight, resins were washed with binding buffer. After resuspension of the pellet in a Sample buffer (Life Technologies) and heating at 100 °C for 3 min, the supernatant was collected by centrifugation at 13,000 × *g* for 20 s for electrophoretic separation in a NuPAGE® gradient gel (Life Technologies). Immunoblotting was conducted using primary antibody (anti-phospho-(Ser) 14-3-3 binding motif antibody (1:1000) or anti-pan 14-3-3 antibody (1:1000) from Cell Signaling Technology), horseradish peroxidase (HRP)-conjugated anti-rabbit IgG (Cell Signaling Technology), and then, an ECL kit for detection of chemiluminescence.

### Cell fractionation

After washing cells with chilled PBS (all subsequent steps, on ice), 200 μl of a lysis buffer (10 mM HEPES, pH 7.9, 1.5 mM MgCl_2_, 10 mM KCl, 0.5 mM DTT, 0.05% Igepal containing protease and phosphatase inhibitors) was added to each well of a 6-well plate. After scraping cells and douncing, the extract was centrifuged at 845 × g for 10 min. Nuclear fraction was resuspended in a buffer containing 5 mM HEPES, pH 7.9, 1.5 mM MgCl_2_, 0.2 mM EDTA, 0.5 mM DTT, 26% glycerol (v/v) and 300 mM NaCl. Both nuclear and cytoplasmic fractions were resuspended in a sample buffer (Life Technologies) for immunoblot analysis.

### RNA extraction and real-time RT-PCR

cDNA was synthesized using total RNA extracted from cells with TRIzol (Life Technologies) and M-MLV Reverse Transcriptase (Promega) according to the manufacturer’s protocol. Real-time RT-PCR was performed using AccuPower® GreenStar^TM^ qPCR master mix (Bioneer) in a QuantStudio3 Real-Time PCR System (Applied Biosystems). All expression values were normalized to *Rpl32* mRNA level. The sequences of primers used for real-time RT-PCR are listed in the Supplementary Tables 1 and 2. Expression of MiTF/TFE family genes in primary mouse islets was studied by RT-PCR using specific primers (*Tfeb* F, GGTCTTGGGCAAATCCCTTC; *Tfeb* R, CATGGCAGCTGTTGGTTCG; *Tfe3* F, CCGTGTTCCTGCTATTGGAA; *Tfe3* R, CGTAGAAGCTGTCAGGATCG; *Mitf* F, GAAGTCGGGGAGGAGTTTCA; *Mitf* R, GCCACTCTCTGTTGCATGA).

### Islet cell death

Primary monkey islets or 1.1B4 cells were placed on a Cell Culture Plate (SPL Life Sciences), and incubated with MSL-7 in the presence or absence of 5 mM 3-MA for 16 h. Apoptosis was quantified by measuring the amount of oligonucleosomes in cell lysate by ELISA employing a kit (Roche Applied Science), according to the manufacturer’s instruction. Apoptosis of wild-type INS-1 cells, *Tfeb*-KO or *Tfe3*-KO INS-1 cells after transfection of *prepro-IAPP-HA* was determined using the same method of oligonucleosome detection.

### Pancreatic insulin content

Pancreatic insulin was extracted and insulin content was measured by ELISA according to the protocol recommended by the manufacturer, and normalized to the total pancreas^38^.

### O_2_ consumption of islets

O_2_ consumption of isolated mouse islets was measured using Seahorse Extracellular Flux (XF^e^96) Analyzer (Agilent Technologies) according to a modification of the manufacturer’s protocol. In brief, islets were seeded into wells of a poly-L-lysine-coated XF96 spheroid microplate (25 islets/well). Islet seeding was done by inserting pipette tip directly over the central depressed chamber into the wells of the spheroid microplate. Islets then were incubated with pre-warmed XF assay medium (Seahorse XF base DMEM medium supplemented with 3 mM glucose, 1% fetal bovine serum, 1 mM sodium pyruvate and 2 mM glutamine) for 1–2 h at 37 °C in a non-CO_2_ incubator. Mitochondrial respiration was measured using the Seahorse extracellular flux analyzer equipped with a spheroid microplate-compatible thermal tray. Basal respiration was first measured in XF assay medium containing 3 mM glucose. Islets were then sequentially exposed to 20 mM glucose, 5 μM oligomycin, 1 μM carbonyl cyanide-4-(trifluoromethoxy)phenylhydrazone (FCCP) and 5 μM rotenone in combination with 5 μM antimycin A (Rot/AA) at the indicated time points to measure glucose-stimulated, ATP-coupled, maximal and non-mitochondrial O_2_ consumption, respectively. O_2_ consumption was analyzed using a software (Wave^TM^, Agilent Technologies).

### Statistical analysis

All values are expressed as the means ± SEM of more than 2 independent experiments. Two-tailed Student’s *t*-test was used to compare values between two groups. One-way ANOVA with Tukey’s test or two-way ANOVA with Bonferroni’s test was employed to compare values between multiple groups. *P* values < 0.05 were considered significant.

### Reporting summary

Further information on research design is available in the Nature Research Reporting Summary linked to this article.

## Supplementary information

Supplementary Information

Reporting Summary

## Data Availability

The data generated or analyzed during this study are available from the corresponding author on reasonable request. Source data are provided with this paper.

## Source data

Source Data
