# Supplementary material for: An autophagy enhancer ameliorates diabetes of human IAPP-transgenic mice through clearance of amyloidogenic oligomer
Source: Nat Commun. 2021 Jan 8;12:183. doi: 10.1038/s41467-020-20454-z (PMC7794419; doi:10.1038/s41467-020-20454-z)
Supplement: Supplementary file 1 — Supplementary Information [file 41467_2020_20454_MOESM1_ESM.pdf]

Supplementary Information

An autophagy enhancer ameliorates diabetes of human *IAPP*-transgenic mice through clearance of amyloidogenic oligomer

Jinyoung Kim, Kihyoun Park, Min Jung Kim, Hyejin Lim, Kook Hwan Kim, Sun-Woo Kim, Eun-Seo Lee, Hyongbum (Henry) Kim, Sung Joo Kim, Kyu Yeon Hur, Jae Hyeon Kim, Jin Hee Ahn, Kun-Ho Yoon, Ji-Won Kim & Myung-Shik Lee

Correspondence to: mslee0923@yuhs.ac

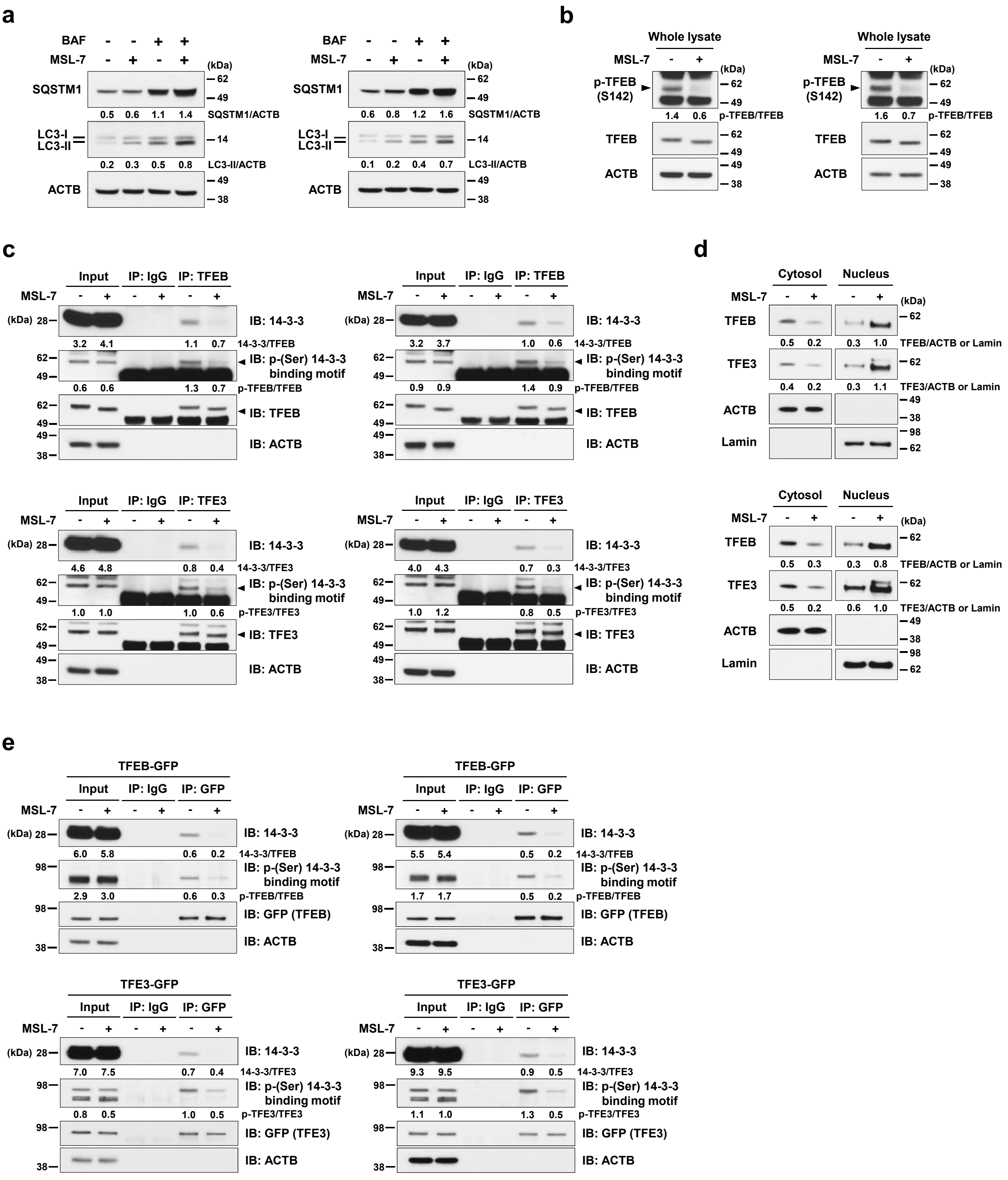

Supplementary Fig. 1 | See next page for caption

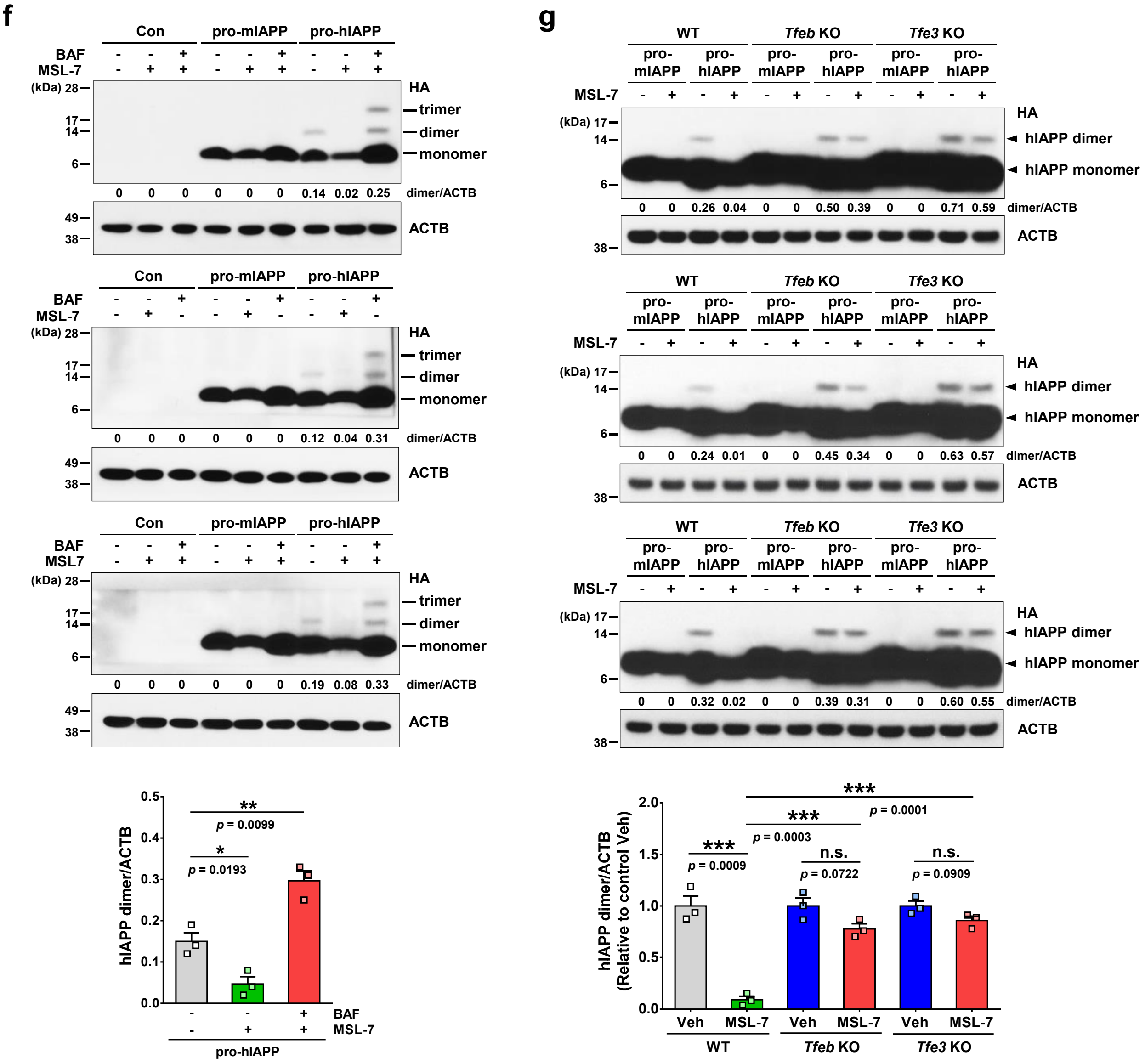

Supplementary Fig. 1 | All immunoblots including representative blots in the Fig. 1, 2, and 4, and quantification of band intensities. a All immunoblots of **Fig. 1b**. **b** All immunoblots of **Fig. 1e**. **c** All immunoblots of **Fig. 1f**. **d** All immunoblots of **Fig. 1g**. **e** All immunoblots of **Fig. 1h**. **f** All immunoblots of **Fig. 2a** with densitometric quantification (F = 35.8, df treatment = 2, df residual = 6). **g** All immunoblots of **Fig. 4c** with densitometric quantification (F = 32.9, df treatment = 5, df residual = 12). All data in this figure are the means ± SEM from 2~3 independent experiments performed. *P, < 0.05; **P, < 0.01; ***P, < 0.001 by one-way ANOVA with Tukey's post-hoc test (**f**,**g**). (n.s., not significant) Source data are provided as a Source Data file.

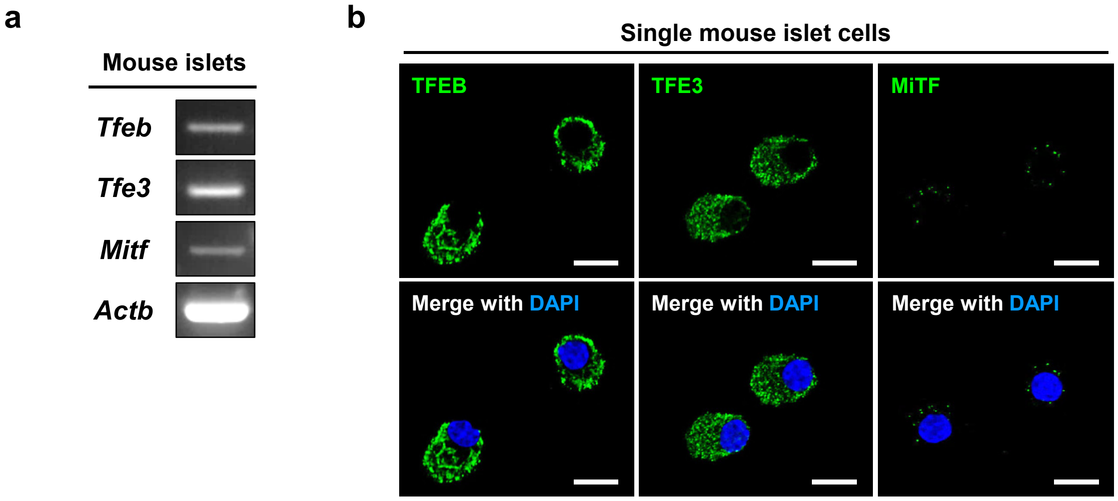

Supplementary Fig. 2 | Expression of MiTF/TFE family member genes in primary murine islets. a RT-PCR was conducted using mRNA prepared from primary murine islet cells and primers specific for the indicated genes. **b** Primary murine islet cells were subjected to immunostaining using anti-TFEB, -TFE3 or -MiTF antibody. (scale bar, 10 μm) Figure in **b** is representative of 2 experiments performed independently.

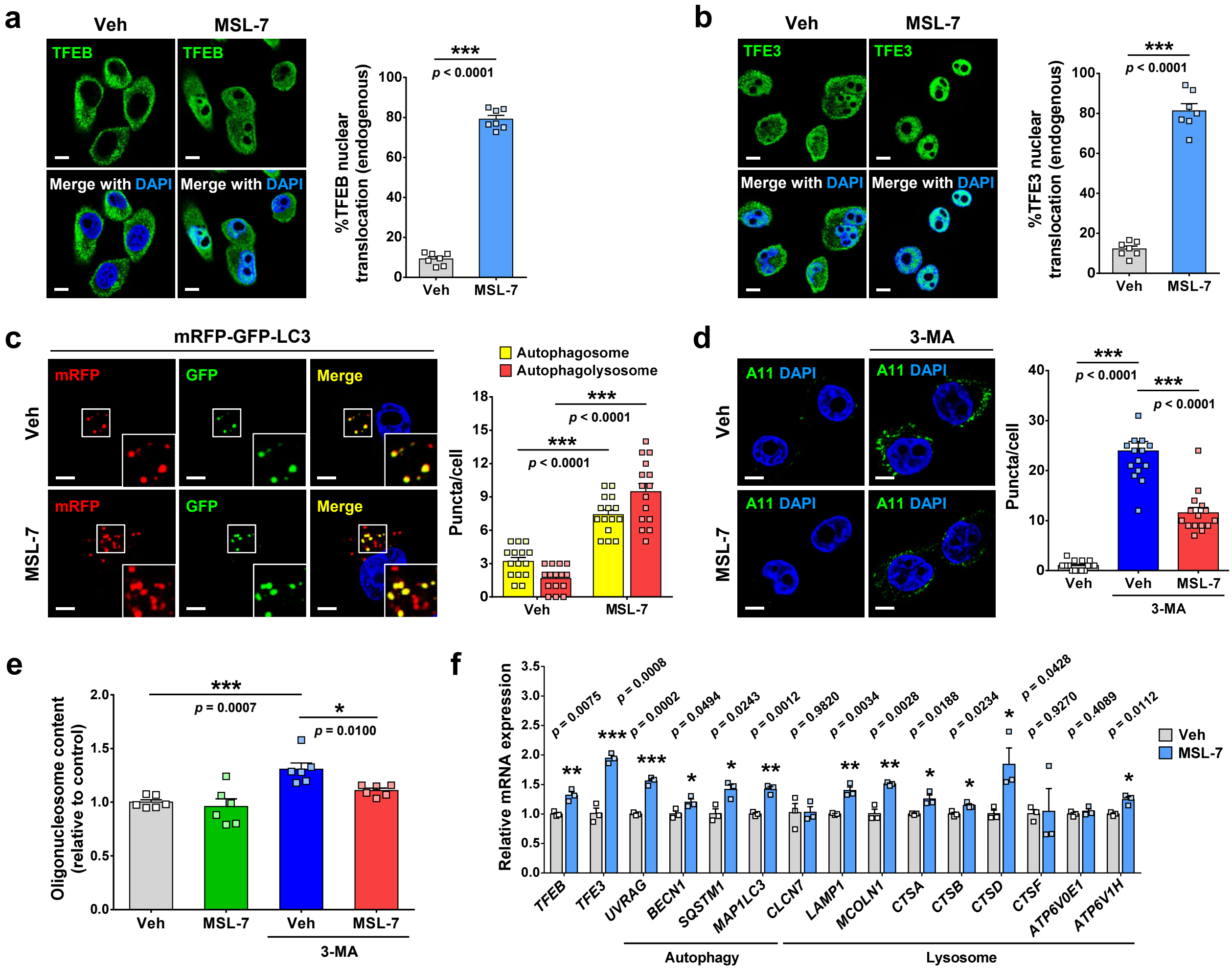

Supplementary Fig 3 | hIAPP oligomer clearance by MSL-7 in a human islet cell line. a-b 1.1B4 cells generated by electrofusion of primary human islet cells to human pancreatic cancer cells were treated with MSL-7 for 4 h, and nuclear translocation of TFEB or TFE3 was examined by confocal microscopy after immunostaining using anti-TFEB (t = 31.7, df = 12) (**a**) or -TFE3 antibodies (t = 18.0, df = 12) (**b**) **c** 1.1B4 cells transfected with *mRFP-GFP-LC3* tandem construct were treated with MSL-7 for 16 h, and the numbers of autophagosome and autophagolysosome were counted (t = 7.5, df = 28 for autophagosome; t = 9.8, df = 28 for autophagolysosome) (right). Representative pictures are presented (left). Inset images were magnified to show red (autophagolysosomes), green, and yellow (autophagosomes) puncta. **d** 1.1B4 cells treated with MSL-7 with or without 3-MA for 16 h were subjected to immunostaining using A11 antibody recognizing hIAPP oligomer. Confocal microscopy was conducted after nuclear staining with DAPI, and the number of A11^{+} puncta was counted (F = 82.8, df treatment = 2, df residual = 42) (right). Representative pictures are presented (left). **e** After treatment of 1.1B4 cells with MSL-7 in the presence or absence of 3-MA, apoptosis was determined (F = 10.2, df treatment = 3, df residual = 20). **f** Real-time RT-PCR was performed using mRNA prepared from 1.1B4 cells that were treated with MSL-7 and primers specific for human autophagy genes or lysosomal genes. All data in this figure are the means ± SEM from more than 3 independent experiments performed in duplicate. (scale bar, 5 μm) *P, < 0.05; **P, < 0.01; ***P, < 0.001 by one-way ANOVA with Tukey's post-hoc test (**d**,**e**) and two-tailed Student's t-test (**a**,**b**,**c**,**f**).

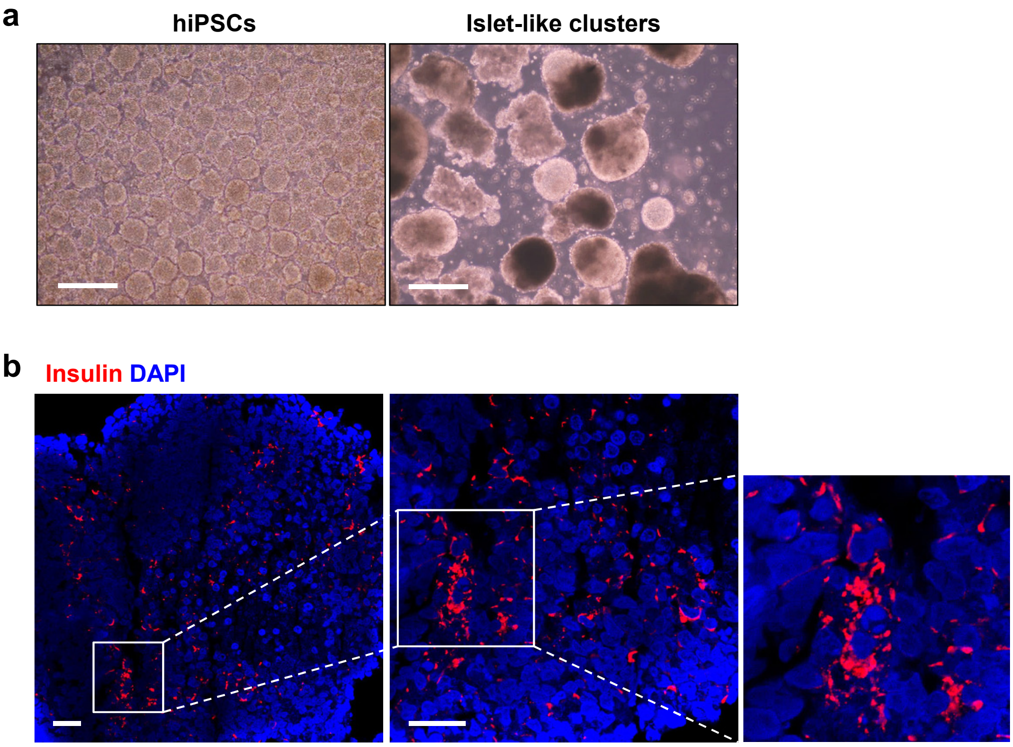

Supplementary Fig. 4 | Generation of hiPSC-derived insulin-producing β-cells. a Human induced pluripotent stem cells (hiPSCs) were differentiated into insulin-producing β-cells (hiPSC-β-cells) using STEMdiff^{TM} Pancreatic Progenitor Kit. Representative pictures of hiPSCs and islet-like clusters including hiPSC-β-cells are shown. (scale bar, 250 μm) **b** hiPSC-β-cells were identified by immunofluorescence of islet-like clusters using anti-insulin antibody. Inset images were magnified. (scale bar, 25 μm) All data in this figure are representative of 2 experiments performed independently.

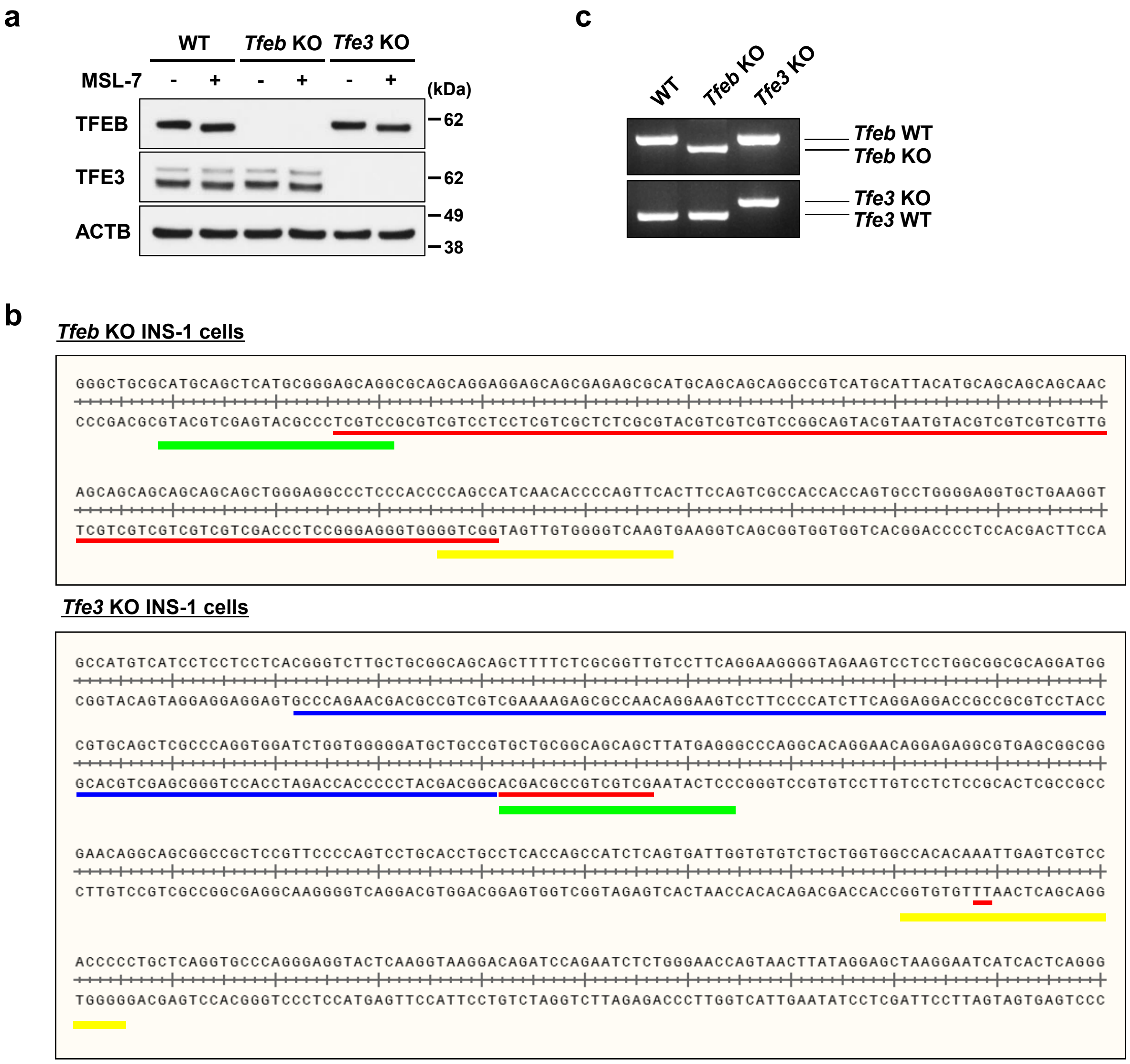

Supplementary Fig. 5 | Generation of *Tfeb*- or *Tfe3*-KO cells. a KO of *Tfeb* or *Tfe3* in INS-1 cells was conducted employing CRISPR/Cas9 technology as described in the Methods. KO of target genes was confirmed by immunoblot analysis using the indicated antibodies. **b** Nucleotide sequencing of target sequences in *Tfeb*- and *Tfe3*-KO cells. Target loci of sgRNA are shown (green underline, respective sgRNA1; yellow underline, respective sgRNA2). Sequences with red underline indicate deleted sequences in *Tfeb*- and *Tfe3*-KO cells. Sequences with blue underline indicate inserted donor sequences in *Tfe3*-KO cells. **c** PCR using cellular DNA and specific primers flanking the target loci. The data in **a** of this figure is representative of 2 experiments performed independently. Source data are provided as a Source Data file.

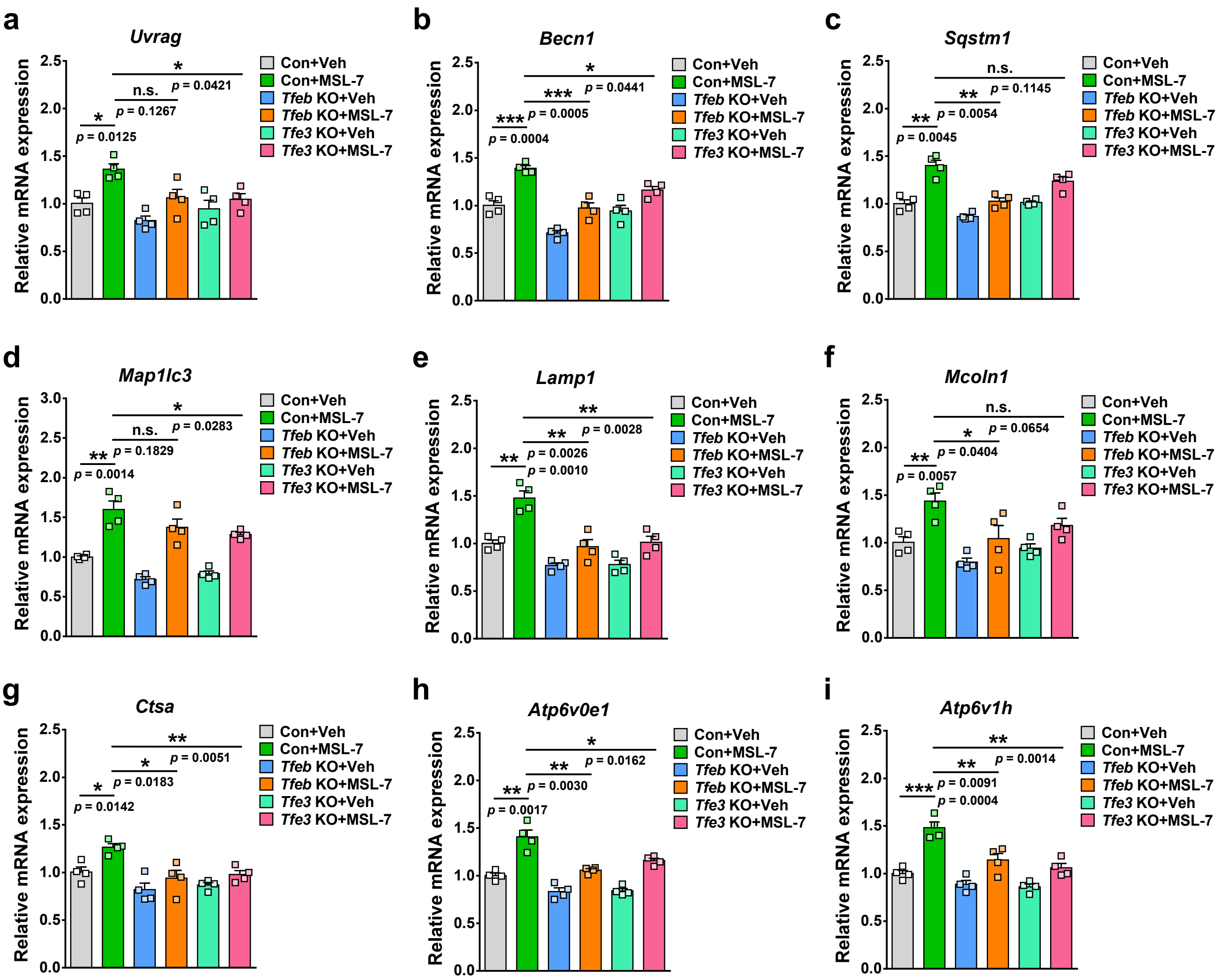

Supplementary Fig. 6 | Reduced induction of autophagy genes and lysosomal genes by MSL-7 in *Tfeb*- or *Tfe3*-KO INS-1 cells. After treatment of *Tfeb*- or *Tfe3*-KO INS-1 cells with MSL-7 for 6 h, real-time RT-PCR was conducted using primers specific for the indicated genes. (F = 6.9, df treatment = 5, df residual = 18 in **a**; F = 27.6, df treatment = 5, df residual = 18 in **b**; F = 26.2, df treatment = 5, df residual = 18 in **c**; F = 28.3, df treatment = 5, df residual = 18 in **d**; F = 21.6, df treatment = 5, df residual = 18 in **e**; F = 7.5, df treatment = 5, df residual = 18 in **f**; F = 8.2, df treatment = 5, df residual = 18 in **g**; F = 32.4, df treatment = 5, df residual = 18 in **h**; F = 22.5, df treatment = 5, df residual = 18 in **i**) All data in this figure are the means ± SEM from more than 3 independent experiments performed in duplicate. *P, < 0.05; **P, < 0.01; ***P, < 0.001 by one-way ANOVA with Tukey's post-hoc test (**a**-**i**). (n.s., not significant)

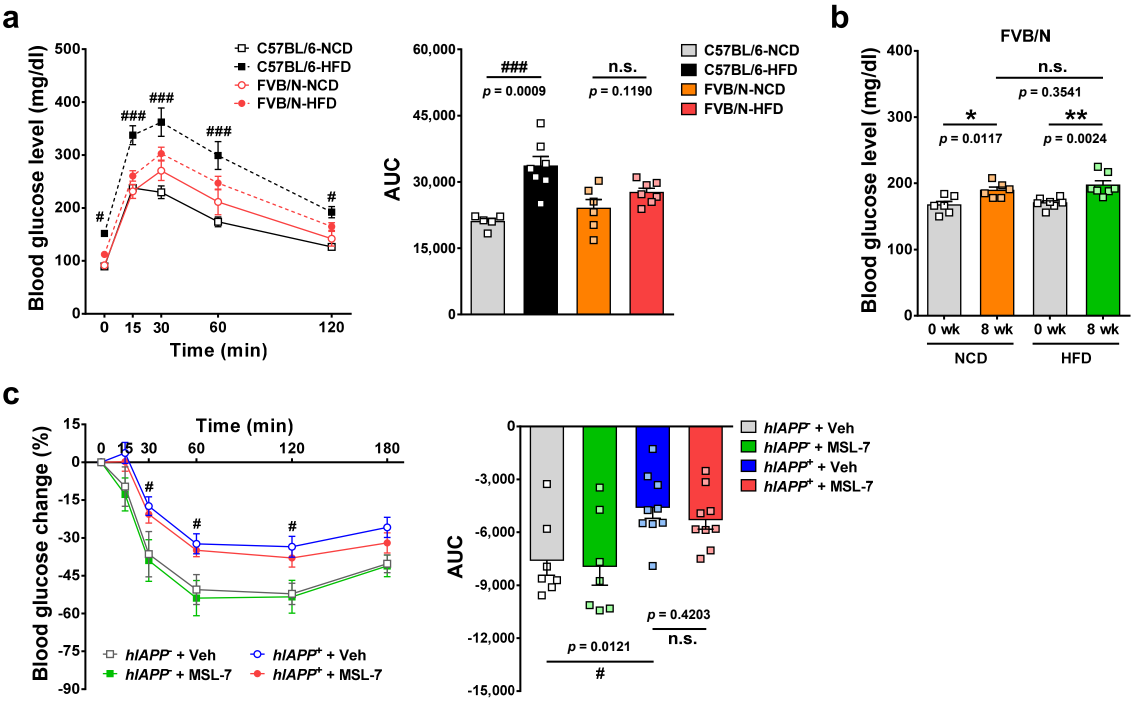

Supplementary Fig. 7 | No effect of MSL-7 on insulin sensitivity of mice of FVB/N background on HFD. a Glucose tolerance test (GTT) was conducted after HFD or NCD feeding of C57BL/6 or FVB/N mice for 8 weeks (F = 13.3, df = 3) (left). Area under the curve (AUC) was calculated (F = 10.0, df treatment = 3, df residual = 21) (right). (n = 5~7) **b** Nonfasting blood glucose level of FVB/N mice after HFD or NCD feeding for 8 weeks was determined (F = 8.5, df treatment = 3, df residual = 22). (n = 6~7) **c** MSL-7 (50 mg/kg) was administered intraperitoneally 3 times a week for 8 weeks to 16-week-old male *hIAPP*$^+$ and *hIAPP*$^-$ mice on HFD, and insulin tolerance test (ITT) was conducted (F = 4.3, df = 3) (left). AUC was calculated (F = 4.7, df treatment = 3, df residual = 28) (right). (n = 7~9) All data in this figure are the means ± SEM from more than 2 independent experiments. $^{\#}P$ or $^{*}P$, < 0.05; $^{**}P$, < 0.01; $^{\#\#\#}P$, < 0.001 by two-way ANOVA with Bonferroni's post-hoc test (**a**,**c**) and one-way ANOVA with Tukey's post-hoc test (**a**-**c**). ($^{\#}$, comparison between HFD-fed C57BL/6 and NCD-fed C57BL/6 mice; *, comparison between HFD-fed FVB/N and NCD-fed FVB/N mice in **a**; $^{\#}$, comparison between Veh-treated *hIAPP*$^+$ and Veh-treated *hIAPP*$^-$ mice in **c**) (n.s., not significant)

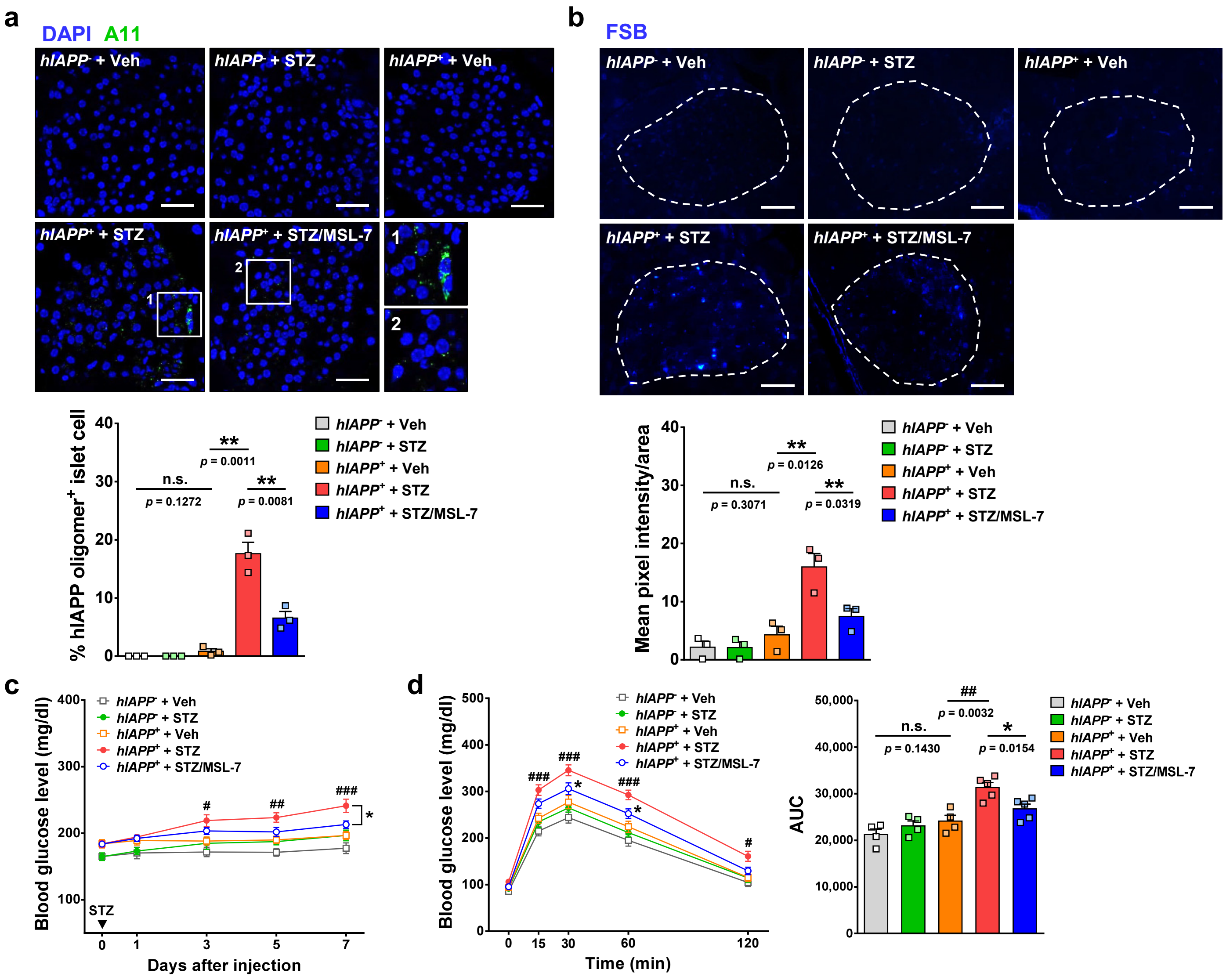

Supplementary Fig. 8 | Effect of autophagy enhancer on hIAPP oligomer accumulation after STZ treatment. **a** After A11 immunostaining of pancreatic sections from STZ-treated *hIAPP*+ and *hIAPP*- mice that were injected with MSL-7 3 times, the percentage of A11 puncta+ cells among total DAPI+ islet cells was determined by confocal microscopy (F = 54.0, df treatment = 4, df residual = 10) (lower). Representative pictures are shown (upper). Inset images were magnified. **b** After FSB staining of pancreatic sections from STZ-treated *hIAPP*+ and *hIAPP*- mice that were injected with MSL-7 3 times, mean fluorescence intensity per islet area was determined (F = 14.9, df treatment = 4, df residual = 10) (lower). Representative pictures are shown (upper). (n = 3, 30~41 islets for **a**,**b**) **c** Nonfasting blood glucose was monitored during MSL-7 administration to STZ-treated mice (F = 11.2, df = 4). **d** GTT was conducted after MSL-7 treatment of STZ-treated mice (F = 10.3, df = 4) (left). AUC was calculated (F = 12.9, df treatment = 4, df residual = 17) (right). (n = 4~5 for **c**,**d**) All data in this figure are the means ± SEM from more than 2 independent experiments. (scale bar, 100 μm) $^{\#}P$ or $^{*}P$, < 0.05; $^{\#\#}P$ or $^{**}P$, < 0.01; $^{\#\#\#}P$, < 0.001 by two-way ANOVA with Bonferroni's post-hoc test (**c**,**d**) and one-way ANOVA with Tukey's post-hoc test (**a**,**b**,**d**). (#, comparison between Veh-treated *hIAPP*+ and STZ-treated *hIAPP*+ mice; *, comparison between STZ/Veh-treated *hIAPP*+ and STZ/MSL-7-treated *hIAPP*+ mice in **c**,**d**) (n.s., not significant)

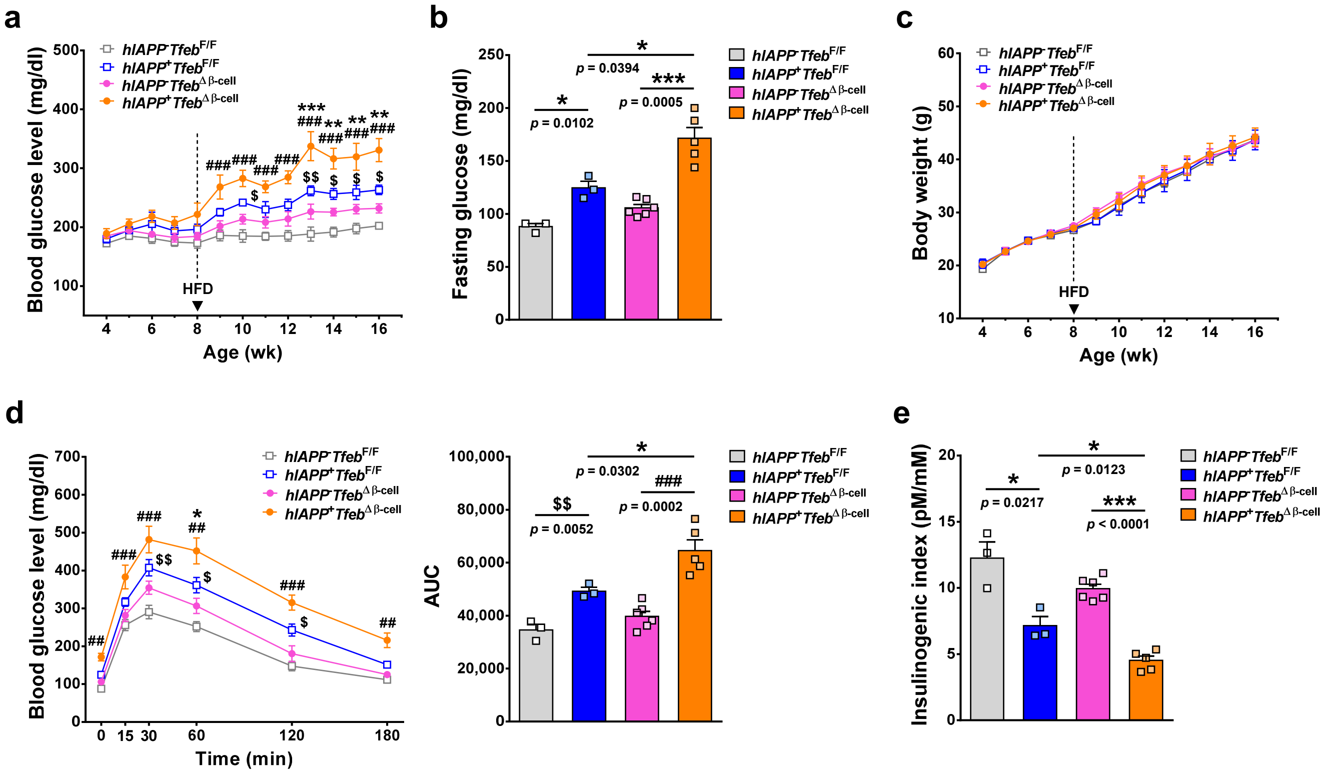

Supplementary Fig. 9 | Effect of pancreatic β-cell-specific *Tfeb* KO on diabetes and β-cell function of *hIAPP*$^{+}$ mice on HFD. a *hIAPP*$^{+}$*Tfeb*$^{\Delta\beta\text{-cell}}$ and *hIAPP*$^{+}$*Tfeb*$^{F/F}$ mice were generated and fed HFD for 8 weeks. Nonfasting blood glucose was monitored weekly (F = 22.6, df = 3). **b** Fasting blood glucose was determined in *hIAPP*$^{+}$*Tfeb*$^{\Delta\beta\text{-cell}}$ and *hIAPP*$^{+}$*Tfeb*$^{F/F}$ mice fed HFD (F = 28.0, df treatment = 3, df residual = 13). **c** Body weight was monitored before and during HFD feeding of *hIAPP*$^{+}$*Tfeb*$^{\Delta\beta\text{-cell}}$ and *hIAPP*$^{+}$*Tfeb*$^{F/F}$ mice. **d** GTT was conducted in *hIAPP*$^{+}$*Tfeb*$^{\Delta\beta\text{-cell}}$ and *hIAPP*$^{+}$*Tfeb*$^{F/F}$ mice fed HFD (F = 12.4, df = 3) (left). AUC was calculated (F = 21.6, df treatment = 3, df residual = 13) (right). **e** Insulinogenic index was calculated in HFD-fed *hIAPP*$^{+}$*Tfeb*$^{\Delta\beta\text{-cell}}$ or *hIAPP*$^{+}$*Tfeb*$^{F/F}$ mice according to the equation described in the Methods (F = 34.0, df treatment = 3, df residual = 13). All data in this figure are the means ± SEM from more than 2 independent experiments. $^{\$}P$ or $^{*}P$, < 0.05; $^{\$\$}P$, $^{**}P$ or $^{\#\#}P$, < 0.01; $^{***}P$ or $^{\#\#\#}P$, < 0.001 by two-way ANOVA with Bonferroni's post-hoc test (**a**,**d**) and one-way ANOVA with Tukey's post-hoc test (**b**,**d**,**e**). ($^{\$}$, comparison between *hIAPP*$^{-}$*Tfeb*$^{F/F}$ and *hIAPP*$^{+}$*Tfeb*$^{F/F}$ mice; *, comparison between *hIAPP*$^{+}$*Tfeb*$^{F/F}$ and *hIAPP*$^{+}$*Tfeb*$^{\Delta\beta\text{-cell}}$ mice; $^{\#}$, comparison between *hIAPP*$^{-}$*Tfeb*$^{\Delta\beta\text{-cell}}$ and *hIAPP*$^{+}$*Tfeb*$^{\Delta\beta\text{-cell}}$ mice in **a**,**d**) (**a**-**e**, n = 3~6)

Supplementary Table 1. Primer sequences of TFEB target genes (rat) for real-time RT-PCR.

Name	Forward (5'→3')	Reverse (5'→3')
Tfeb	GGTCTTGGGCAAATCCCTTC	ATCCTCGGAGTCTTTAAGCG
Tfe3	CATGCAACTGAGACAGCTCG	GCTGTCAGGATCAAGGATGT
Uvrag	GCTCCATTTGAACACAAGGC	ATTGCACACTGGGCTCTATG
Becn1	CCCAGCCAGGATGATGTCTAC	GTATCTGTGCATTCCTCACACA
Sqstm1	TGGGAACTCGCTATAAGTGC	GGGAAAGATGAGCTTGCTGT
Map1lc3	GTTAACATGAGCGAGTTGGT	GTTTCTTGGGAGGCATAGACC
Clcn7	GGCAACCTGACGAGGAGA	GAGCTTCTCGTTGTGTGGAA
Lamp1	CTCGCAATCACCTTTGGAGA	GCCTTGATGTCAGTTGTGGA
Mcoln1	GGCGCGGCTCAGAGA	CATCAACTTGCAGGGCTTG
Ctsa	TATCTTCACTCGCTTGCCAC	GAGCCTTCCGAACATATGGG
Ctsb	CCAATGGCCGAGTCAATGTG	GGAGGGATGGTGTAGGGTAAG
Ctsd	GGAAAGACGATCTGCCTGAG	GCAAAGCCGACCCTATTGTA
Ctsf	ATGTTGGACAAGGATCCCCT	GGTGATCCCATACTGAGCTG
Atp6v0e1	CTTTGATCGTGATGAGCGTG	TCCAAACAGAGGATTGAGCTG
Atp6v1h	CCAGGACCTTAGAATCTTGACA	AGAAGAGCATTGTAGCGGAC
Rpl32	GCTGCTGATGTGCAACAAAT	TTCATTCTCTTCGCTGCGT

Supplementary Table 2. Primer sequences of TFEB target genes (human) for real-time RT-PCR.

Name	Forward (5'→3')	Reverse (5'→3')
TFEB	CGCATCAAGGAGTTGGGAAT	GAGCTGCTTGTTGGTCATCT
TFE3	AATATCACTGCAGGCCACAC	CAGCAAGACCCTCGATGAAG
UVRAG	GACTAATGGTCAGGTGTGACAG	GTCCTTTGTCTGGTGAAGGGAT
BECN1	AGCTGCCGTTATACTGTTCTG	AAACGTGTCTCGCCTTTCTC
SQSTM1	GATTCGCCGCTTCAGCTT	CGTCCTCATCGCGGTAGT
MAP1LC3	GACCGCTGTAAGGAGGTACA	GACCAACTCGCTCATGTTGA
CLCN7	CAACGTCTCTAAGAAGGTGTCC	GACTCGGAAAAGCGCAGAAC
LAMP1	TGCAAGTTCTAGCCGGTTTT	TGTAGGAATTGCCGACTGTG
MCOLN1	CCGATGGTGGTTACTGACTG	CAGCTTGTGGAATTTGAGCG
CTSA	CCTGGAGTACAACCCCTATTC	AGGCGGAAGAAATCTTGAAGG
CTSB	GAGAATGGCACACCCTACTG	GCCACCACTTCTGATTCGAT
CTSD	ACAAGTTCACGTCCATCCG	GGGCGTCCATGTAGTTCTTG
CTSF	CTTCGCGCTGGAGATGTTC	GAGTACAGCGACCCCTGA
ATP6V0E1	TTGTGATGAGCGTGTTCTGG	AGCCAAAAGAGATAGCAGCA
ATP6V1H	GTCTCTTCAAGTGATAGTTCGC	GATACTGGAGCTGAAAGCCAC
RPL32	TGCCCAACATTGGTTATGGAA	AACATTGTGAGCGATCTCGG
