# Supplementary material for: An autophagy enhancer ameliorates diabetes of human IAPP-transgenic mice through clearance of amyloidogenic oligomer
Source: Nat Commun. 2021 Jan 8;12:183. doi: 10.1038/s41467-020-20454-z (PMC7794419; doi:10.1038/s41467-020-20454-z)
Supplement: Supplementary file 2 — Reporting Summary [file 41467_2020_20454_MOESM2_ESM.pdf]

nature research

Corresponding author(s): Myung-Shik Lee

Last updated by author(s): Nov 6, 2020

Reporting Summary

Nature Research wishes to improve the reproducibility of the work that we publish. This form provides structure for consistency and transparency in reporting. For further information on Nature Research policies, see our Editorial Policies and the Editorial Policy Checklist.

Statistics

For all statistical analyses, confirm that the following items are present in the figure legend, table legend, main text, or Methods section.

n/a	Confirmed	
☐	☒	The exact sample size (*n*) for each experimental group/condition, given as a discrete number and unit of measurement
☐	☒	A statement on whether measurements were taken from distinct samples or whether the same sample was measured repeatedly
☐	☒	The statistical test(s) used AND whether they are one- or two-sided<br>*Only common tests should be described solely by name; describe more complex techniques in the Methods section.*
☒	☐	A description of all covariates tested
☐	☒	A description of any assumptions or corrections, such as tests of normality and adjustment for multiple comparisons
☐	☒	A full description of the statistical parameters including central tendency (e.g. means) or other basic estimates (e.g. regression coefficient) AND variation (e.g. standard deviation) or associated estimates of uncertainty (e.g. confidence intervals)
☐	☒	For null hypothesis testing, the test statistic (e.g. *F*, *t*, *r*) with confidence intervals, effect sizes, degrees of freedom and *P* value noted<br>*Give P values as exact values whenever suitable.*
☒	☐	For Bayesian analysis, information on the choice of priors and Markov chain Monte Carlo settings
☒	☐	For hierarchical and complex designs, identification of the appropriate level for tests and full reporting of outcomes
☒	☐	Estimates of effect sizes (e.g. Cohen's *d*, Pearson's *r*), indicating how they were calculated

Our web collection on statistics for biologists contains articles on many of the points above.

Software and code

Policy information about availability of computer code

Data collection	GraphPad Prism 6 software, Zen2009 software, ImageJ software, QuantStudio Design & Analysis software, Wave software, SnapGene software
Data analysis	Statistics and Graphs were performed using GraphPad Prism 6 software (Ver. 6.01).<br>Fluorescent confocal images were exported using Zen2009 software (Ver. 2009 Light edition).<br>Band intensity of immunoblot, mean pixel intensity of fluorescence per area were quantified using ImageJ software (Ver. 1.46r).<br>Analysis of gene expression level was run and exported using QuantStudio Design & Analysis software (Ver. 1.5.1).<br>Oxygen consumption was analyzed using Wave software (Ver. 2.3.0.19).<br>Gemonic DNA sequencing of CRISPR/Cas9 Tfeb- or Tfe3-KO was analyzed using SnapGene Viewer software (Ver. 5.0.4).

For manuscripts utilizing custom algorithms or software that are central to the research but not yet described in published literature, software must be made available to editors and reviewers. We strongly encourage code deposition in a community repository (e.g. GitHub). See the Nature Research guidelines for submitting code & software for further information.

Data

Policy information about availability of data

All manuscripts must include a data availability statement. This statement should provide the following information, where applicable:

- Accession codes, unique identifiers, or web links for publicly available datasets
- A list of figures that have associated raw data
- A description of any restrictions on data availability

The data sets generated in the current study are available from the corresponding author on reasonable request.

Field-specific reporting

Please select the one below that is the best fit for your research. If you are not sure, read the appropriate sections before making your selection.

- [x] Life sciences
- [ ] Behavioural & social sciences
- [ ] Ecological, evolutionary & environmental sciences

For a reference copy of the document with all sections, see nature.com/documents/nr-reporting-summary-flat.pdf

Life sciences study design

All studies must disclose on these points even when the disclosure is negative.

Sample size	For animal experiments, the sample size was determined based on similar previous studies of our laboratory and on previous experiments using similar methodologies. Detailed sample size were described in the figure legends.
Data exclusions	The samples for both in vivo and in vitro experiments were not excluded from all the analyses.
Replication	All findings of this study were reproducible reliably. All data in this manuscript are from at least more than 2 independent experiments.
Randomization	For animal experiments, age-matched male mice were randomly assigned to experimental groups.
Blinding	The investigators were not blinded to allocation during experiments and outcome assessment. However, to minimize any potential bias, all male mice were randomly assigned to experimental groups or control groups (age-matched). All samples were collected and analyzed under the same conditions.

Reporting for specific materials, systems and methods

We require information from authors about some types of materials, experimental systems and methods used in many studies. Here, indicate whether each material, system or method listed is relevant to your study. If you are not sure if a list item applies to your research, read the appropriate section before selecting a response.

Materials & experimental systems

n/a	Involved in the study	
	x	Antibodies
	x	Eukaryotic cell lines
x		Palaeontology and archaeology
	x	Animals and other organisms
	x	Human research participants
x		Clinical data
x		Dual use research of concern

Methods

n/a	Involved in the study	
x		ChIP-seq
x		Flow cytometry
x		MRI-based neuroimaging

Antibodies

Antibodies used

The following antibodies were used for immunoblotting (IB), immunoprecipitation (IP), immunofluorescence (IF), immunohistochemistry (IHC):
1) SQSTM1 (Progen Biotechnik, GP62-C, IB 1:1,000 dilution),
2) LC3 (Novus Biologicals, NB100-2331, IB 1:1,000 dilution or MBL M152-3, IF 1:150 dilution),
3) β-actin[C4] (ACTB; Santa Cruz Biotechnology, SC-47778, IB 1:4000 dilution),
4) TFEB (Bethyl Laboratory, A303-673A, IF 1:150, IP 1:1,000, IB 1:1,000 dilution),
5) TFEB (Proteintech, 13372-1-AP, IF 1:150 dilution),
6) TFE3 (Sigma Aldrich, HPA023881, IF 1:150, IP 1:1,000, IB 1:1,000 dilution),
7) MiTF[C5] (Thermo Fisher Scientific, MA5-14146, IF 1:150 dilution),
8) Phospho-S142-TFEB (Merk Millipore, ABE1971, IB 1:1,000 dilution),
9) Phospho-(Ser) 14-3-3 binding motif (Cell Signaling Technology, #9601, IB 1:1,000 dilution),
10) 14-3-3 (pan) (Cell Signaling Technology, #8312, IB 1:1,000 dilution),
11) Lamin A[4A58] (Santa Cruz Biotechnology, SC-71481, IB 1:1000 dilution),
12) GFP (AbFrontier LF-PA0043, IP 1:1,000, IB 1:1,000 dilution),
13) HA[C29F4] (Cell Signaling Technology, #3724, IB 1:1,000 dilution),
14) A11 (Merk Millipore, AB9234, Lot # LV1403351, IF 1:150 dilution),
15) Insulin (Cell Signaling Technology, #4590, IHC 1:150 dilution),
16) Insulin[L6B10] (Cell Signaling Technology, #8138, IF 1:150 dilution),
18) Alexa 488-anti-mouse IgG (H+L) (Invitrogen, A11001, Lot # 2127435, IF 1:200 dilution),
18) Alexa 488-anti-rabbit IgG (H+L) (Invitrogen, A11008, Lot # 2110498, IF 1:200 dilution),

19) Alexa 594-anti-mouse IgG (H+L) (Invitrogen, A11005, Lot # 210496, IF 1:200 dilution),
20) Alexa 568-anti-rabbit IgG (H+L) (Invitrogen, A11011, Lot # 2088069, IF 1:200 dilution),
21) Biotinylated anti-rabbit IgG (H+L) (Vector, BA-1000, IHC 1:100 dilution)

Validation

The information above can also be found in the Methods section. For all antibodies, additional information on application, species cross-reactivity or related publications can be found on the manufacturer's website.

(Applications / Species Cross-Reactivity / Source)

1) SQSTM1 (Progen Biotechnik, GP62-C): IB (1:1,000-1:2,000), IHC (1:100-1:200) / Human, Mouse, Rat / Guinea pig
2) LC3 (Novus Biologicals, NB100-2331): IB (2μg/ml), ICC/IF (1:100-1:300), IHC (1:200-1:400), IHC-Fr, IHC-P (1:200-1:400), IP (20μg per 500μg of lysate) / Human, Mouse, Rat, Zebrafish / Rabbit
3) β-actin[C4] (ACTB; Santa Cruz Biotechnology, SC-47778): IB (1:100-1:1,000), IP (1-2 μg per 100-500 μg of total protein), IF (1:50-1:500), IHC (1:50-1:500), ELISA (1:30-1:3,000) / Mouse, Rat, Human, Avian, Bovine, Canine, Porcine, Rabbit / Mouse
4) TFEB (Bethyl Laboratory, A303-673A): IB (1:2,000-1:10,000), IP (2 to 10μg per mg lysate), IHC-P (1:500-1:2,000), IF (1:250-1:1,000) / Human, Mouse / Rabbit
5) TFEB (Proteintech, 13372-1-AP): IB (1:500-1:2,000), IP (0.5-4μg), IF (1:20-1:500), IHC (1:50-1:200) / Human, Mouse, Rat / Rabbit
6) TFE3 (Sigma Aldrich, HPA023881): IB (1:100-1:250), IF (1-4μg per mL), IHC (1:500-1:1,000) / Human, Mouse, Rat / Rabbit
7) MiTF[C5] (Thermo Fisher Scientific, MA5-14146): IB (1:100-1:1,000), ICC/IF (1:50), IHC (1:20-1:500) / Human, Mouse, Rat / Mouse
8) Phospho-S142-TFEB (Merk Millipore, ABE1971): IB (1:12,500) / Human (Predicted to react with Mouse, Rat) / Rabbit
9) Phospho-(Ser) 14-3-3 binding motif (Cell Signaling Technology, #9601): IB (1:1,000), IP (1:50), IHC (1:50), ELISA (1:1,000) / All / Rabbit
10) 14-3-3 (pan) (Cell Signaling Technology, #8312): IB (1:1000) / Human, Mouse, Rat, Monkey, Bovine, Pig / Rabbit
11) Lamin A[4A58] (Santa Cruz Biotechnology, SC-71481): IB (1:100-1:100), IP (1-2μg per 100-500μg of total protein), IF (1:50-1:500), ELISA (1:30-1:3,000) / Human, Mouse, Rat / Mouse
12) GFP (AbFrontier LF-PA0043, IP 1:1,000): IB (1:1,000-5,000), IP (1-2μl) / - / Rabbit
13) HA[C29F4] (Cell Signaling Technology, #3724): IB (1:1,000), IP (1:50), IHC-P (1:800), ICC/IF (1:3,200), ChIP (1:50) / All / Rabbit
14) A11 (Merk Millipore, AB9234, Lot # LV1403351): IB (1:500), IF(1:200), IHC (1:1,000-1:10,000), IP (1:1,000) / Human, Mouse, Rat / Rabbit
15) Insulin (Cell Signaling Technology, #4590): IHC-P (1:100), ICC/IF (1:100) / Human, Mouse, Rat / Rabbit
16) Insulin[L6B10] (Cell Signaling Technology, #8138): IB (1:1,000), IP (1:50), IF (1:800) / Rat (Predicted to react with Human, Mouse) / Mouse

*Immunoblotting (IB), immunoprecipitation (IP), immunofluorescence (IF), immunocytochemistry (ICC), immunohistochemistry (IHC)

Eukaryotic cell lines

Policy information about cell lines

Cell line source(s)

INS-1 insulinoma cells were kindly provided by Dr. C. Wollhein, University of Geneva, Switzerland.
Primary mouse islet cells and monkey islet cells were isolated from C57BL/6 mice and Cynomolgus monkeys, respectively.
1.1B4 cells were obtained from the European Collection of Authenticated Cell Cultures (ECACC) through Fadzilah Adibah Abdul Majid, Universiti Malaysia Terengganu.
Human induced pluripotent stem cell-derived β-cells (hiPSC-β-cells) were generated using STEMdiff Pancreatic Progenitor Kit.
Tfeb- or Tfe3-KO INS-1 cells were generated by using CRISPR/Cas9 technology.

Authentication

INS-1 and 1.1B4 cells were not authenticated but displayed expected morphology.
hiPSC-β-cells were authenticated by immunofluorescence of islet-like clusters using anti-insulin antibody for human.
Tfeb- or Tfe3-KO INS-1 cells were authenticated by immunobloting using antibodies against TFEB, TFE3 and nucleotide sequencing of target sequences.

Mycoplasma contamination

All cells were negative for mycoplama contamination, except primary mouse islet cells and monkey islet cells. Primary mouse islet cells and monkey islet cells were not tested for mycoplama contamination.

Commonly misidentified lines
(See ICLAC register)

No commonly misidentified cell lines were used.

Animals and other organisms

Policy information about studies involving animals; ARRIVE guidelines recommended for reporting animal research

Laboratory animals

hIAPP+ mice were purchased from Jackson Laboratory (FVB/N-Tg (Ins2-IAPP) RHFSoel/J mice, Stock No. 008232).
TfebF/F mice were generated by breeding Tfebtm1a(EUCOMM)Wtsi mice (Mutant Mouse Resource and Research Center, MMRRC) with FLPeR mice (Wellcome Trust Sanger Institute).
Rip-Cre mice were purchased from Jackson Laboratory.
C57BL/6 mice were purchased from Orientbio Laboratory (Korea).
As a diet-induced diabetic model, 8-week-old mice were fed HFD (60% kcal fat, Research Diet #D12492) for 8 weeks. Male mice with age range 16-18 weeks were used in all experiments.
Cynomolgus monkeys (male; age, 56 months) were purchased from Guangxi Grandforest Scientific Primate Company Ltd. and were maintained in the Orientbio Animal Facility.

Wild animals	This study did not involve wild animals.
Field-collected samples	This study did not involve field collected samples.
Ethics oversight	All mice experiments were approved by the Institutional Animal Care and Use Committee of Yonsei University Health System (IACUC of YUHS) and were conducted in accordance with the guidelines of the IACUC of YUHS.<br>Monkey experiments were approved by the IACUC of Orientbio, another AAALAC-accredited unit.

Note that full information on the approval of the study protocol must also be provided in the manuscript.

Human research participants

Policy information about studies involving human research participants

Population characteristics	Sex: Male.<br>Age: - (This study used a human cord blood mononuclear cell sources).<br>We accessed the HLA-type CBMC library of the Catholic Hematopoietic Stem Cell Bank of Korea.<br>Through the CBMC bank, we were able to find the homozygous HLA types. HLA type (HLA-A, HLA-B, HLA-DR combined) of hiPS cells of this study was HLA-A*11, HLA-B*15, HLA-DR1*04.
Recruitment	We screened and prepared cord blood mononuclear cells, from the database of the Catholic Hematopoietic Stem Cell Bank of Korea.<br>We analyzed about 30,000 donated cord blood mononuclear cells to find cells with homogygous HLA-types. Finally, we generated 9 iPS cells lines. HLA-type did not affect efficiency of hiPSC-β-cell generation.
Ethics oversight	The use of human materials including human iPSCs was approved by the Seoul St Mary's Hospital Institutional review board, The Catholic University of Korea (approval No. KC16TISI0981).

Note that full information on the approval of the study protocol must also be provided in the manuscript.
