# Supplementary material for: An autophagy enhancer ameliorates diabetes of human IAPP-transgenic mice through clearance of amyloidogenic oligomer
Source: Nat Commun. 2021 Jan 8;12:183. doi: 10.1038/s41467-020-20454-z (PMC7794419; doi:10.1038/s41467-020-20454-z)

Source Data
(Uncropped blots and gels)

# An autophagy enhancer ameliorates diabetes of human *IAPP*-transgenic mice through clearance of amyloidogenic oligomer

Jinyoung Kim, Kihyoun Park, Min Jung Kim, Hyejin Lim, Kook Hwan Kim, Sun-Woo Kim, Eun-Seo Lee, Hyongbum (Henry) Kim, Sung Joo Kim, Kyu Yeon Hur, Jae Hyeon Kim, Jin Hee Ahn, Kun-Ho Yoon, Ji-Won Kim & Myung-Shik Lee

Correspondence to: mslee0923@yuhs.ac

## Fig. 1b & Supplementary Fig. 1a (right)

**IB: SQSTM1 (Progen, 1:5000)**

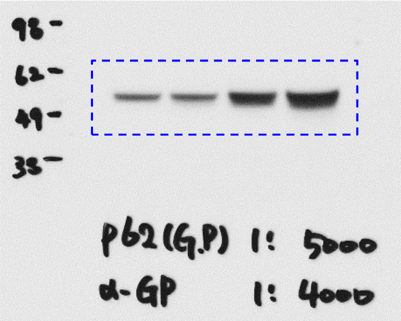


**IB: LC3 (Novus, 1:1000)**

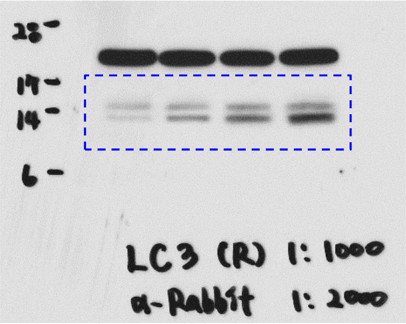


**IB: ACTB (Santa Cruz, 1:4000)**

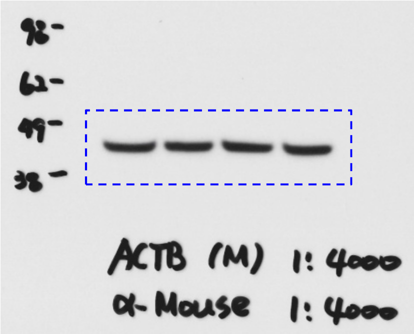


**IB: SQSTM1 (Progen, 1:5000)**

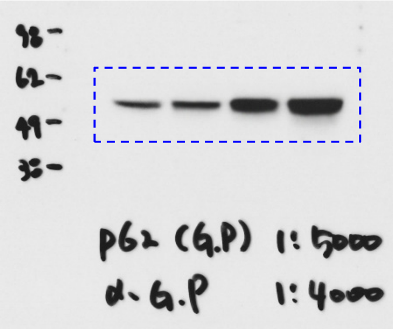


**IB: LC3 (Novus, 1:1000)**

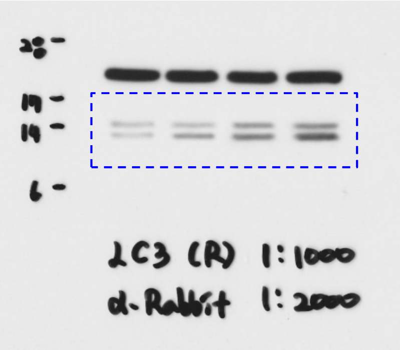


**IB: ACTB (Santa Cruz, 1:4000)**

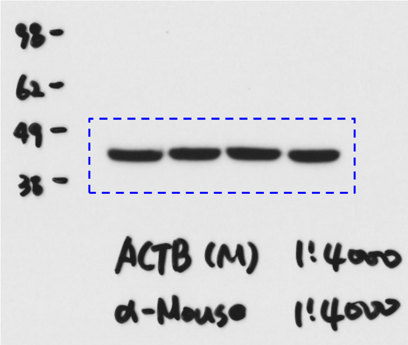

## Fig. 1e & Supplementary Fig. 1b (right)

**IB: p-TFEB (Millipore, 1:2000)**

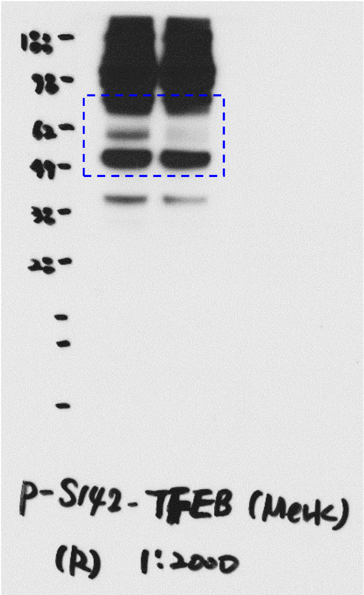


**IB: TFEB (Bethyl, 1:2000)**

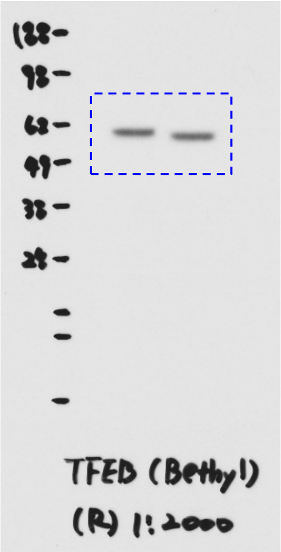


**IB: ACTB (Santa Cruz, 1:4000)**

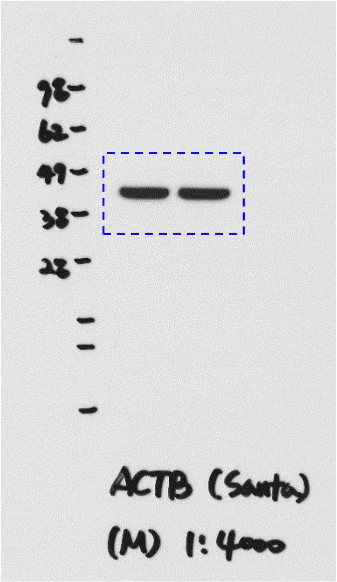


**IB: p-TFEB (Millipore, 1:2000)**

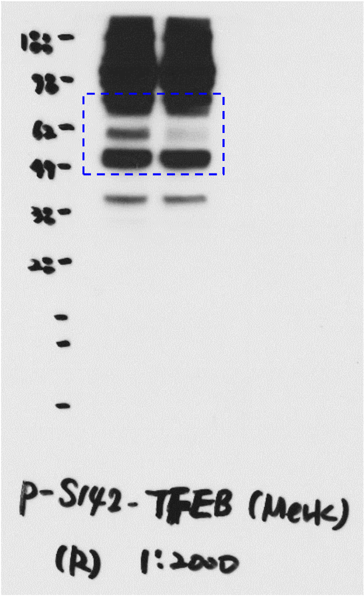


**IB: TFEB (Proteintech, 1:2000)**

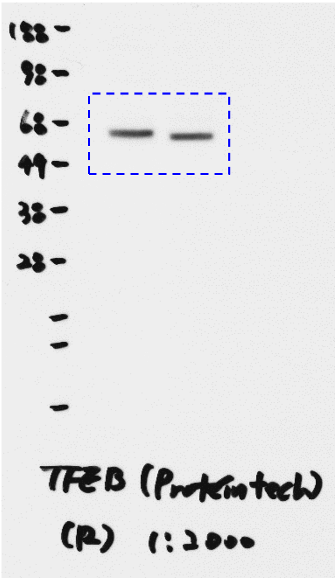


**IB: ACTB (Santa Cruz, 1:4000)**

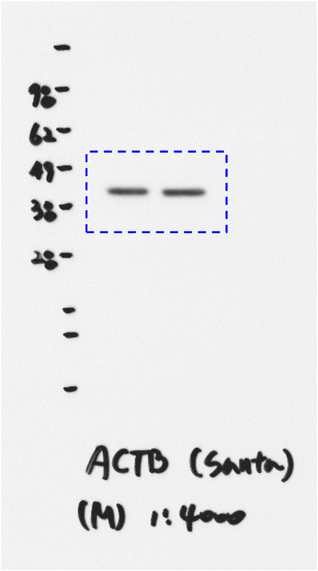

## Fig. 1f (left) & Supplementary Fig. 1c (upper)

**IB: 14-3-3 (Cell signaling, 1:1000)**

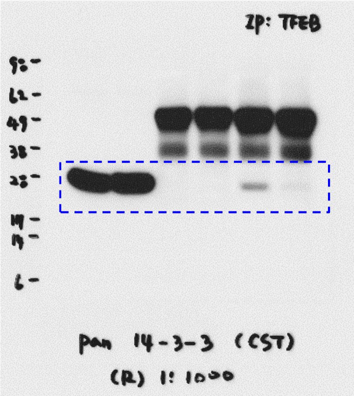


**IB: p-(Ser) 14-3-3 binding Motif (Cell signaling, 1:1000)**

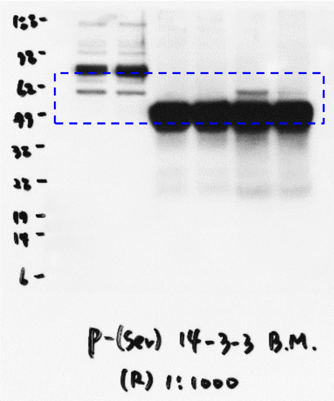


**IB: TFEB (Bethyl, 1:2000)**

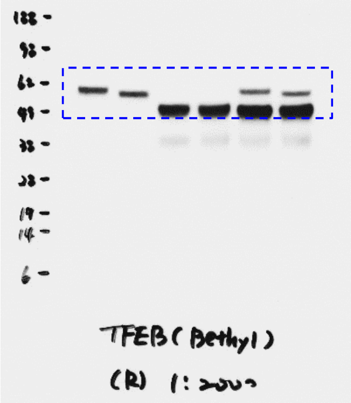


**IB: ACTB (Santa Cruz, 1:4000)**

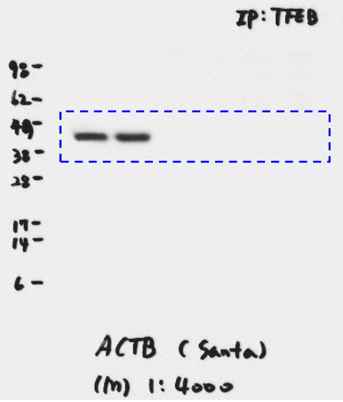


**IP: TFEB (Bethyl, 1:1000, 1μg/ml lysate)**

**IB: 14-3-3 (Cell signaling, 1:1000)**

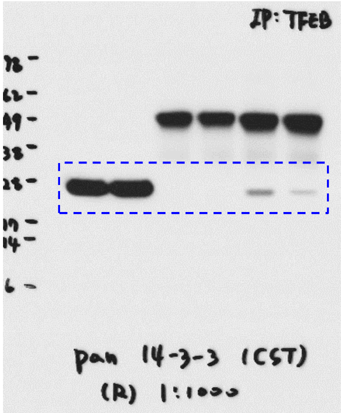


**IB: p-(Ser) 14-3-3 binding Motif (Cell signaling, 1:1000)**

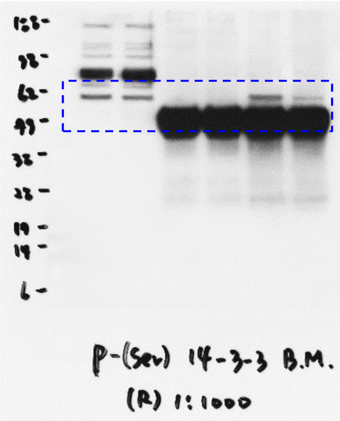


**IB: TFEB (Bethyl, 1:2000)**

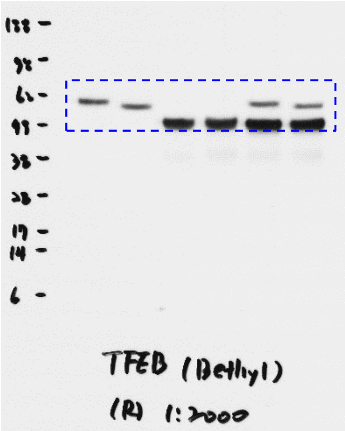


**IB: ACTB (Santa Cruz, 1:4000)**

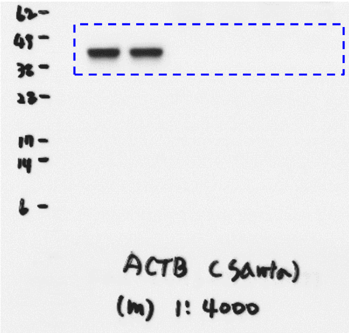


**IP: TFEB (Bethyl, 1:1000, 1μg/ml lysate)**

## Fig. 1f (right) & Supplementary Fig. 1c (lower)

IB: 14-3-3
(Cell signaling, 1:1000)

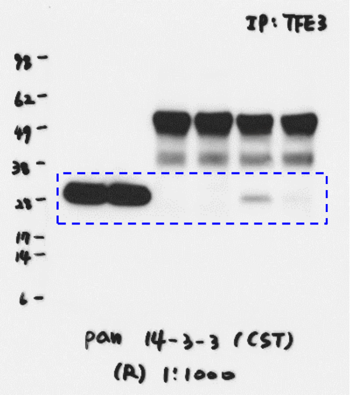


IB: p-(Ser) 14-3-3 binding
Motif (Cell signaling, 1:1000)

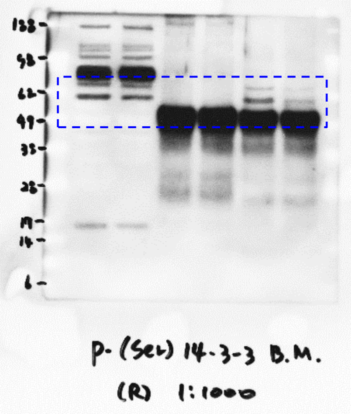


IB: TFE3
(Sigma, 1:2000)

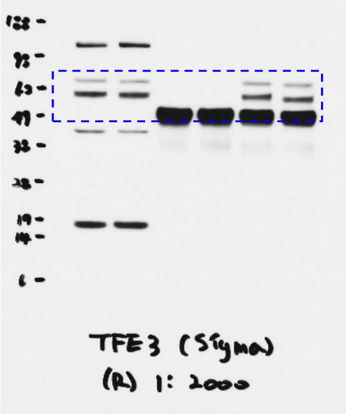


IB: ACTB
(Santa Cruz, 1:4000)

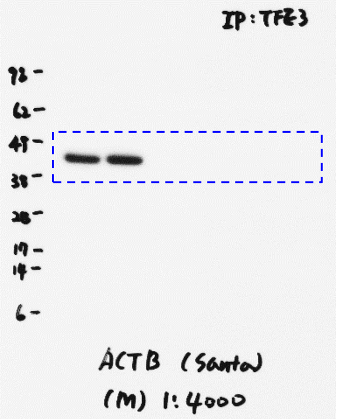


IP: TFE3 (Sigma, 1:1000, 1μg/ml lysate)

IB: 14-3-3
(Cell signaling, 1:1000)

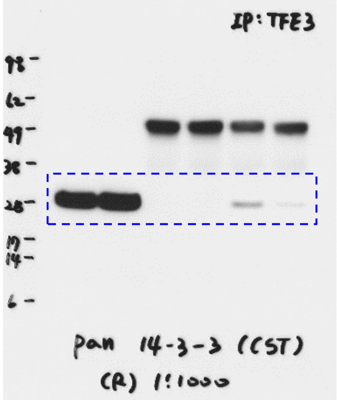


IB: p-(Ser) 14-3-3 binding
Motif (Cell signaling, 1:1000)

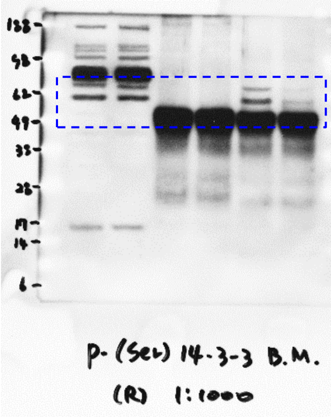


IB: TFE3
(Sigma, 1:2000)

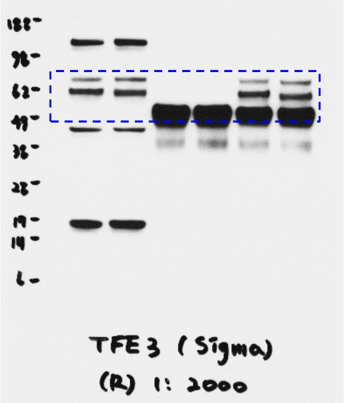


IB: ACTB
(Santa Cruz, 1:4000)

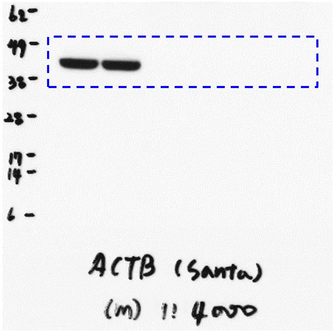


IP: TFE3 (Sigma, 1:1000, 1μg/ml lysate)

## Fig. 1g & Supplementary Fig. 1e (lower)

IB: TFEB (Bethyl, 1:1000)

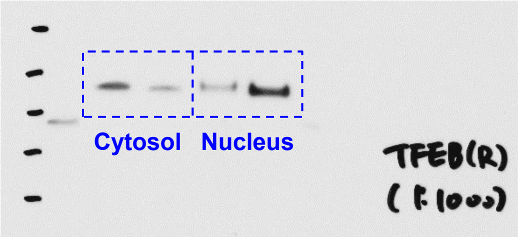


IB: TFEB (Bethyl, 1:1000)

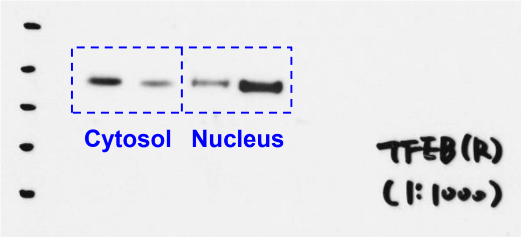


IB: TFE3 (Sigma, 1:1000)

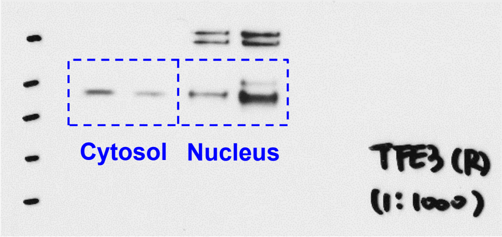


IB: TFE3 (Sigma, 1:1000)

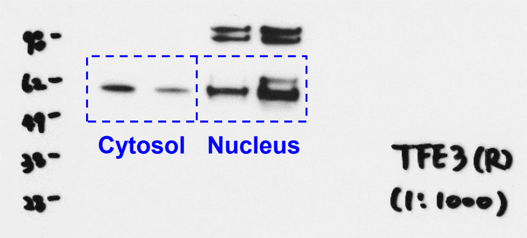


IB: ACTB
(Santa Cruz, 1:4000)

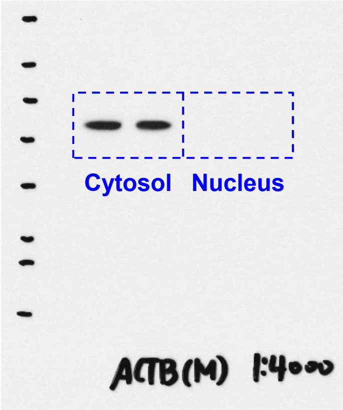


IB: ACTB
(Santa Cruz, 1:4000)

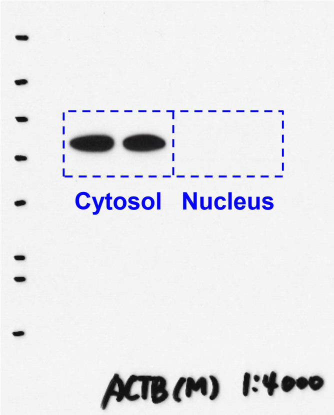


IB: Lamin
(Santa Cruz, 1:2000)

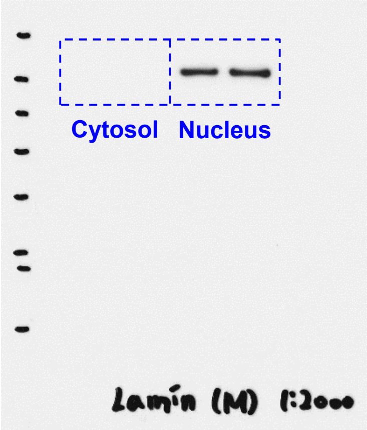


IB: Lamin
(Santa Cruz, 1:2000)

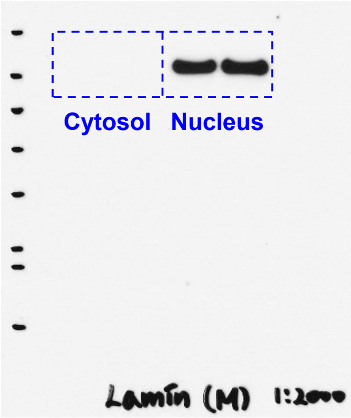

## Fig. 1h (left) & Supplementary Fig. 1e (upper)

**IB: 14-3-3 (Cell signaling, 1:1000)**

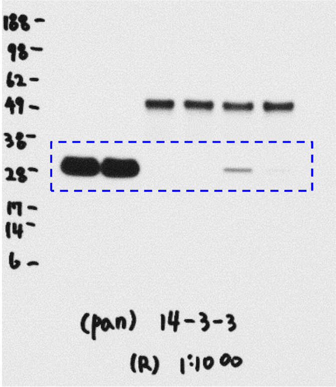


**IB: p-(Ser) 14-3-3 binding Motif (Cell signaling, 1:1000)**

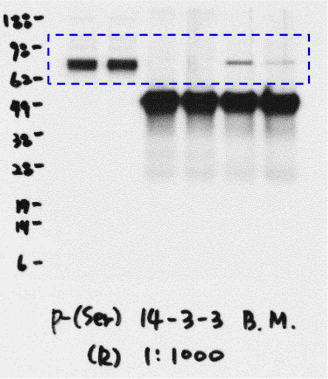


**IB: GFP (AbFrontier, 1:1000)**

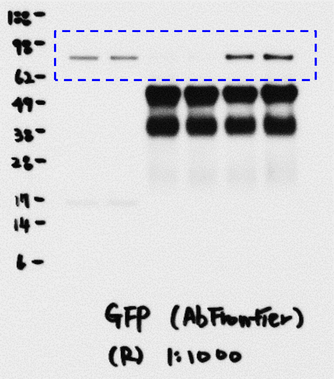


**IB: ACTB (Santa Cruz, 1:4000)**

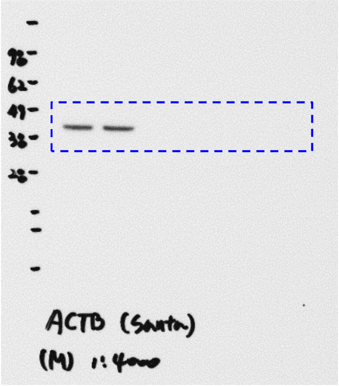


**IP: GFP (AbFrontier, 1:1000, 1μg/ml lysate)**

**IB: 14-3-3 (Cell signaling, 1:1000)**

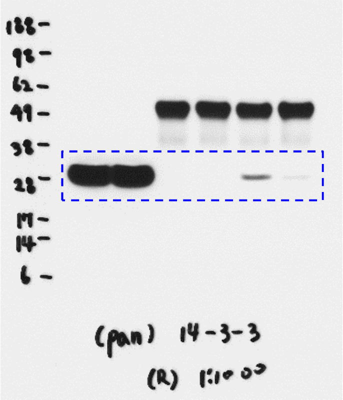


**IB: p-(Ser) 14-3-3 binding Motif (Cell signaling, 1:1000)**

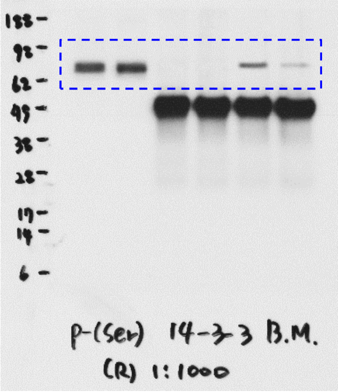


**IB: GFP (AbFrontier, 1:1000)**

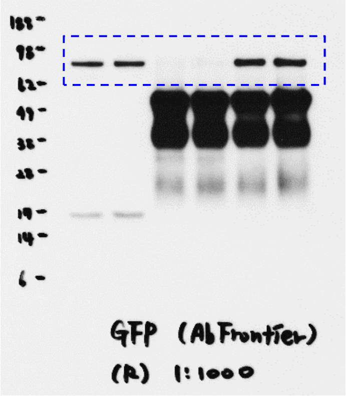


**IB: ACTB (Santa Cruz, 1:4000)**

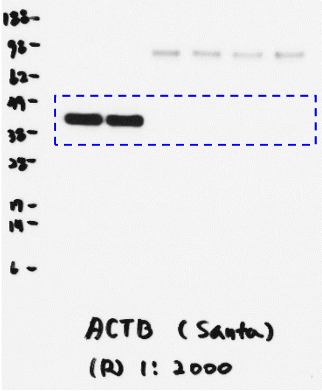


**IP: GFP (AbFrontier, 1:1000, 1μg/ml lysate)**

## Fig. 1h (right) & Supplementary Fig. 1e (lower)

IB: 14-3-3
(Cell signaling, 1:1000)

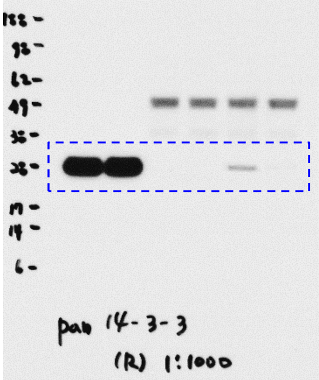


IB: p-(Ser) 14-3-3 binding
Motif (Cell signaling, 1:1000)

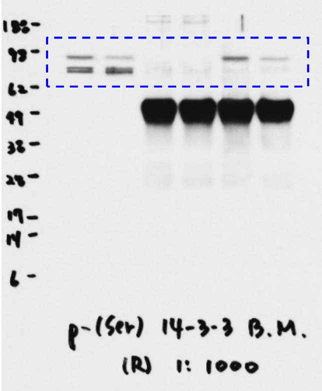


IB: GFP
(AbFrontier, 1:1000)

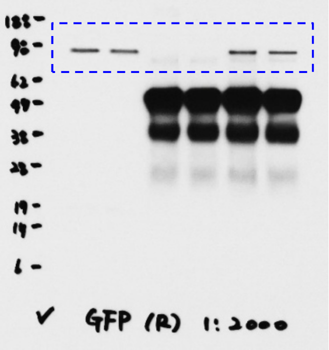


IB: ACTB
(Santa Cruz, 1:4000)

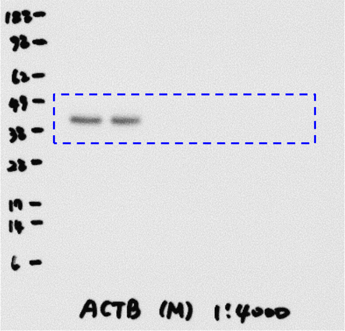


IP: GFP (AbFrontier, 1:1000, 1μg/ml lysate)

IB: 14-3-3
(Cell signaling, 1:1000)

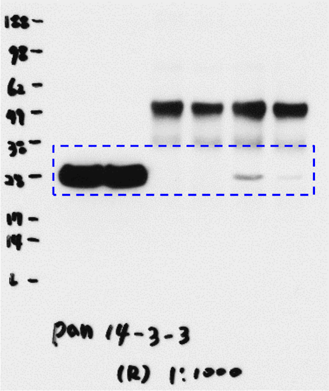


IB: p-(Ser) 14-3-3 binding
Motif (Cell signaling, 1:1000)

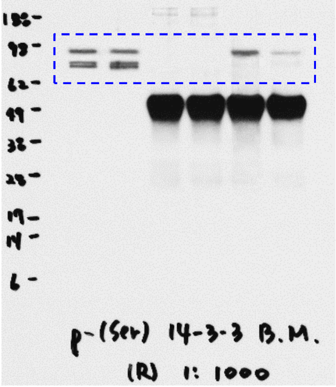


IB: GFP
(AbFrontier, 1:1000)

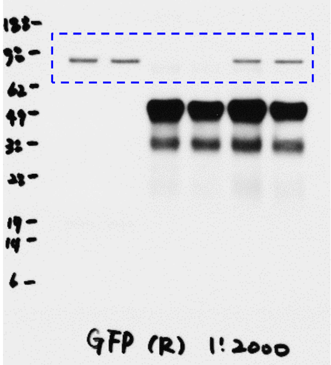


IB: ACTB
(Santa Cruz, 1:4000)

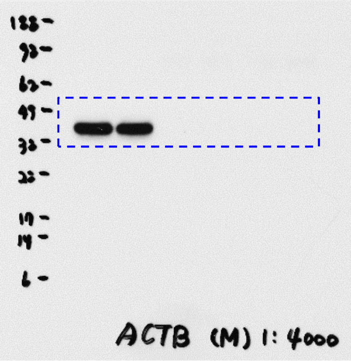


IP: GFP (AbFrontier, 1:1000, 1μg/ml lysate)

## Fig. 2a & Supplementary Fig. 1f (lower)

**IB: HA (Cell signaling, 1:2000)**

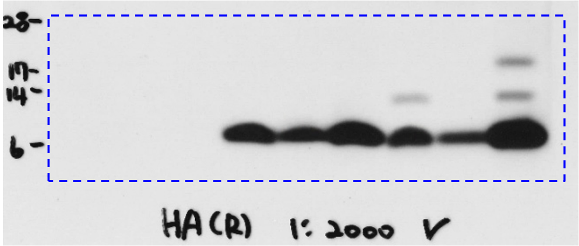


**IB: ACTB (Santa Cruz, 1:8000)**

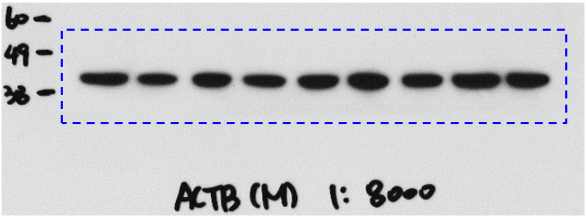


**IB: HA (Cell signaling, 1:2000)**

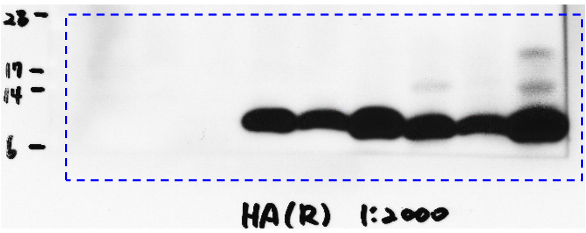


**IB: ACTB (Santa Cruz, 1:4000)**

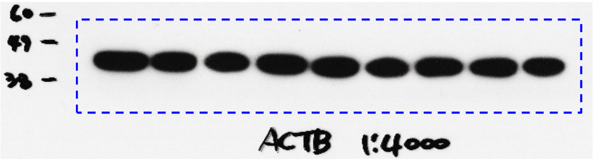


**IB: HA (Cell signaling, 1:2000)**

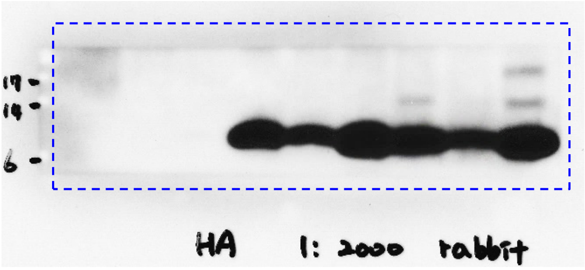


**IB: ACTB (Santa Cruz, 1:4000)**

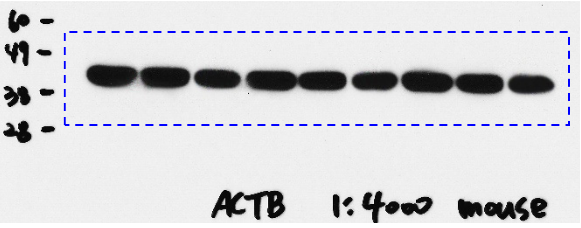

## Fig. 4c & Supplementary Fig. 1g (lower)

IB: HA (Cell signaling, 1:1000)

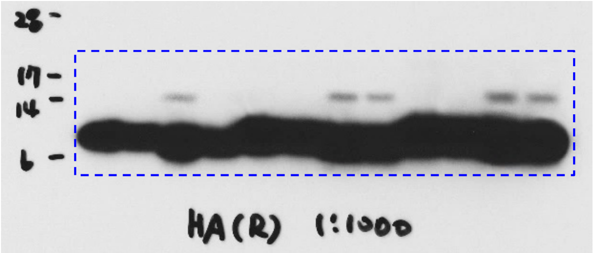


IB: ACTB (Santa Cruz, 1:2000)

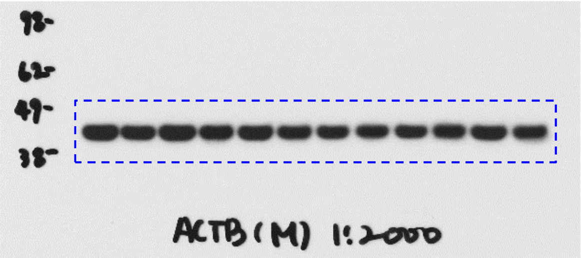


IB: HA (Cell signaling, 1:1000)

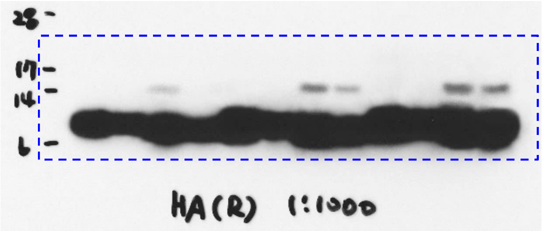


IB: ACTB (Santa Cruz, 1:4000)

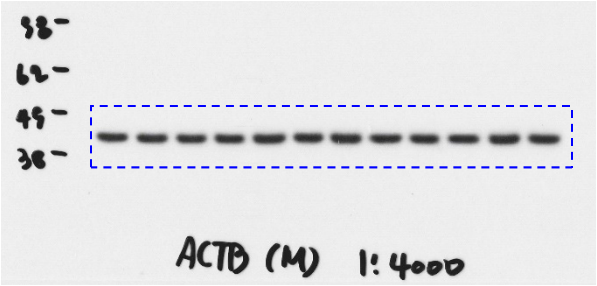


IB: HA (Cell signaling, 1:1000)

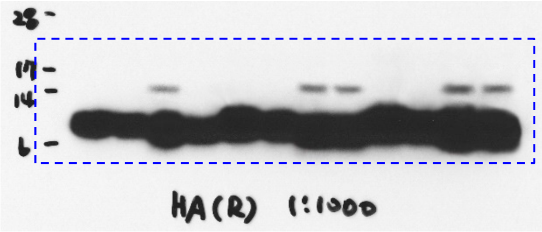


IB: ACTB (Santa Cruz, 1:1000)

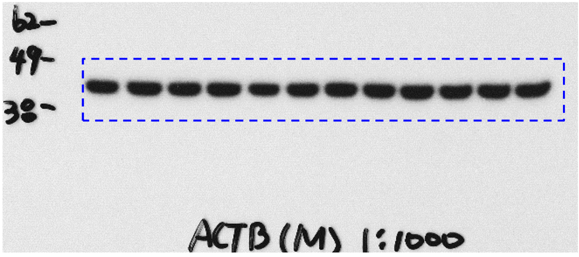

## Supplementary Fig. 5a

IB: TFEB (Bethyl, 1:1000)

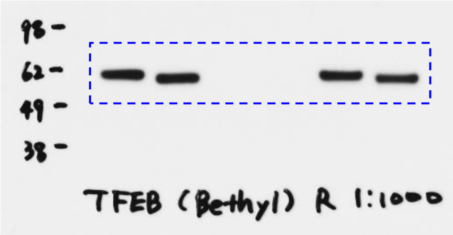


IB: TFEB (Bethyl, 1:1000)

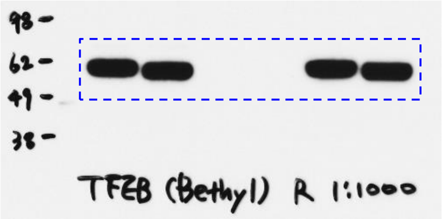


IB: TFE3 (Sigma, 1:1000)

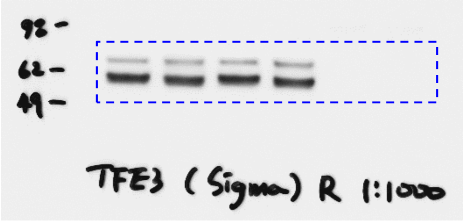


IB: TFE3 (Sigma, 1:1000)

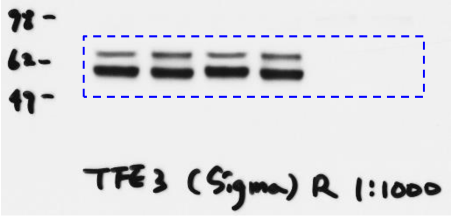


IB: ACTB (Santa Cruz, 1:2000)

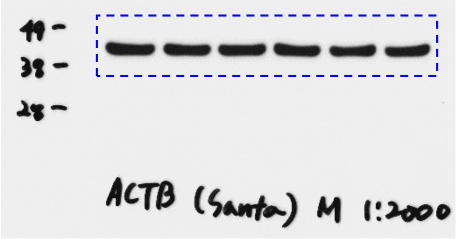


IB: ACTB (Santa Cruz, 1:2000)

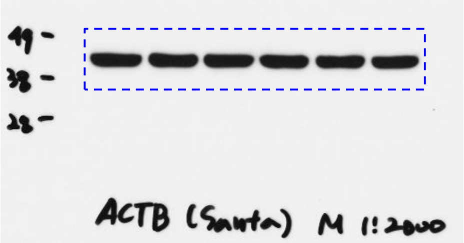

Supplement: Supplementary file 3 — Source Data [file 41467_2020_20454_MOESM3_ESM.zip › Final2_Nat.Commun-04-Source Data (Uncropped blots and gels)_PDF.pdf]
